# Primary healthcare and child and maternal health in the Middle East and North Africa (MENA): A retrospective analysis of 29 national survey data from 13 countries

**DOI:** 10.1016/j.ssmph.2021.100727

**Published:** 2021-01-12

**Authors:** Zlatko Nikoloski, Hrayr Wannis, Leonardo Menchini, Anirban Chatterjee

**Affiliations:** a Department of Health Policy, London School of Economics and Political Science, United Kingdom; b UNICEF Regional Office for the Middle East and North Africa, Jordan

**Keywords:** MENA, Primary healthcare, Equity of access, Maternal and child health

## Abstract

The objective of this paper is three-fold: (i) to analyse the coverage and equity of access to selected maternal and child healthcare interventions, particularly those delivered in Primary Healthcare (PHC) setting; (ii) to analyse the main drivers of inequitable access to selected interventions; and (iii) to synthesise and compare the results across the Middle East and North Africa (MENA) region as well as over time. We analysed data for five key maternal and child healthcare interventions from 29 national surveys (DHS and MICS) covering 13 MENA countries and spanning a period of almost 20 years (2000–2018). We calculated coverage indicators, concentration indices (CI) and decomposition of CIs according to standard definitions. We synthetized the results by country groups based on their human development index (HDI). Over time and among countries that started from a lower base, there has been an improvement in coverage and equity of selected interventions (four ante-natal care visits and skilled birth assistance). When considering the place of skilled delivery, there is a clear rich-poor divide, with women from richer wealth quintiles gravitating toward private healthcare facilities and those from poorer wealth quintiles toward public ones. While most of the care-seeking for common child illnesses occurs in PHC facilities, a fraction (20–30 percent) of care-seeking takes place in secondary healthcare facilities. PHC has played a role in improving coverage and equity of access in key maternal and child health interventions in the wider MENA region. Better integration of care, strengthening and improving the PHC network could increase the use of cost-effective interventions, which are key to improving maternal and child health.

## Highlights

• Over time, there has been an increase in the coverage of selected interventions delivered in PHC setting coupled with an increase in equity of access.
• Except in the low HDI countries, there is clear split indicating pro-rich inequity in delivery at private secondary healthcare facilities and pro-poor inequity in delivery at public ones.
• A fraction of care (about 20–30 percent) for common childhood illnesses takes place in secondary healthcare facilities.
• Richer households are more likely to seek care for the common child illnesses in private healthcare facilities (primary and secondary) while poorer ones are more likely to go in public healthcare facilities.

## Introduction

Middle East and North Africa (MENA) is a heterogeneous region that encompasses countries at various level of socio-economic development (World Development Indicators, 2020). Over the last couple of decades, the countries in the region have made significant strides in improving both, maternal and child health on their path towards meeting the health related SDGs (World Development Indicators, 2020). Nevertheless, there are significant inequities in child and maternal health outcomes mainly driven by disparities in access to child and maternal healthcare interventions (UNICEF, 2019).

One way to address some of these inequities in access to maternal and child healthcare interventions is by strengthening the role of the primary healthcare (PHC). Most services recommended for the continuum of maternal and child care can be delivered at the PHC level, with effective links to community-based services and referral systems to secondary and tertiary levels of care (Save the Children, 2016). Ultimately, PHC becomes even more attractive in resource constrained settings considering the Universal Health Coverage agenda (WHO, 2018). Moreover, PHC could play a role in averting the indirect impact of COVID-19 by restoring communities’ trust in the health system.

The heterogeneity in the level of socio-economic development in the MENA countries influences the overall development of PHC with a few commonalities that emerge across the countries in the region. First, except for the Gulf countries, the countries in MENA continue to be low investors in healthcare (including PHC), with a high share of out-of-pocket payment in total health spending – exceeding 75% in some of the region's low-income countries (World Health Organization, 2017). Second, the overall orientation of the healthcare system towards curative, rather than preventative care, results not only with a skewed distribution of the overall workforce but also with underinvestment in PHC (Kronfol, 2012). Third, and closely related to the previous point, there is not enough focus on the family practice system with shortages of trained human resources in family medicine (Agreus & Saab, 2015). Fourth, except for a few countries, there is a lack of community-based approaches in the process of strengthening PHC (van Weel et al., 2018). More specifically, there has been a lack of systemic approach to the community-based activities as well as a lack of incentives for community volunteers, resulting with a small effect on health outcomes, particularly for disadvantaged groups (Rashad, 2011). Finally, there is a segmentation in terms of provision of healthcare, with large share of utilization taking place in the private sector. However, the range of services provided in the private sector vary, standards are variable, regulation is poor and there is insufficient information about the financial burden to the users. More importantly, private care providers are often reluctant to invest in preventive care, particularly in remote or deprived areas (World Health Organization, 2015).

This structure and organization of PHC in the wider region has implications on the overall access to healthcare and, more specifically, on the access to maternal and child health relevant interventions. Against this background, building on the importance of PHC in the continuum of maternal and child healthcare and relying on 29 datasets from 13 MENA countries, the objective of this paper is three-fold: (i) to analyse the coverage and equity of access to selected maternal and child healthcare interventions, particularly those delivered in PHC setting; (ii) to analyse the main drivers of inequitable access to selected interventions; and (iii) to synthesise and compare the results across the region. In doing so, we group the countries in four major groups, based on their HDI (human development index): (a) very high HDI countries; (b) high HDI countries; (c) medium HDI countries; and (d) low HDI countries.

## Methods

### Data sources and indicators

In doing the analysis, we relied on all available MICS and DHS surveys for the countries of the MENA region since the early 2000s (Appendix Table A1; note the surveys for 2011 and 2018 for Morocco are national health surveys though they broadly follow the DHS/MICS structure). The definitions of the indicators used in our analysis are presented in Table 1, which includes a standard set of indicators used in assessing progress towards SDGs. Some of them (e.g. place of delivery, place of seeking care for common childhood illness) were selected so as to focus the analysis on interventions that could be cost-effectively delivered in PHC. It is important to note that while both, DHS and MICS surveys include similar information in terms of the place where care for various interventions was sought (e.g. primary vs. secondary, public vs. private) some harmonization across countries was needed (see the Supplementary material).

**Table 1:** Indicator definitions.

Indicator name	Indicator description	Numerator	Denominator
Antenatal care (4 or more visits)	Percent of women (counted for each pregnancy) attended at least four times during pregnancy by any provider (skilled or unskilled) for reasons related to the pregnancy in the two years (MICS) or five years (DHS) prior to the survey	Number of women (counted for each pregnancy) attended at least four times during pregnancy by any provider (skilled or unskilled) for reasons related to the pregnancy in the two years (MICS) or five years (DHS) prior to the survey	Total number of women (counted for each pregnancy) between 15 and 49 years who had a live birth in the last two years prior to the survey in the case of MICS (or five in the case of DHS)
Place of delivery	Percent of women with live births in the last two years (MICS) or last five years (DHS) delivered in the following settings: (i) at home; (ii) in public primary healthcare facilities; (iii) in private primary healthcare facilities; (iv) in public secondary healthcare facilities; and (v) in private secondary healthcare facilities	Number of women with live births in the two years (MICS) or five years (DHS) prior to the survey delivered in the following five settings: (i) at home; (ii) in public primary healthcare facilities; (iii) in private primary healthcare facilities; (iv) in public secondary healthcare facilities; and (v) in private secondary healthcare facilities	Total number of women aged 15–49 years who had a live birth in the last two years prior to the survey in the case of MICS (or five in the case of DHS)
Skilled birth assistance (SDG indicator 3.1.2)	Percent of women with live births in the last two years (MICS) or last five years (DHS) attended by skilled health personnel (doctor, nurse or a midwife)	Number of women with live births in the two years prior to the survey (MICS) or last five years (DHS) attended during delivery by skilled attendants (doctor, nurse or a midwife)	Total number of women aged 15–49 years who had a live birth in the last two years prior to the survey in the case of MICS (or five in the case of DHS)
Healthcare seeking for diarrhoea	Percent of children aged 0 to 5 who sought care for diarrhoea in the following facilities: (i) public primary healthcare facility; (ii) private primary healthcare facility; (iii) public secondary healthcare facility; and (iv) private secondary healthcare facility.	Number of children with diarrhoea symptoms over the last two week who have sought care in the following facilities: (i) public primary healthcare facility; (ii) private primary healthcare facility; (iii) public secondary healthcare facility; and (iv) private secondary healthcare facility.	Total number of children aged 0–5 years of age with symptoms of diarrhoea over the last two weeks.
Healthcare seeking for cough/difficulty breathing^a^	Percent of children aged 0 to 5 who sought care for cough/difficulty breathing in the following facilities: (i) public primary healthcare facility; (ii) private primary healthcare facility; (iii) public secondary healthcare facility; and (iv) private secondary healthcare facility.	Number of children with cough/difficulty breathing symptoms over the last two weeks who have sought care in the following facilities: (i) public primary healthcare facility; (ii) private primary healthcare facility; (iii) public secondary healthcare facility; and (iv) private secondary healthcare facility.	Total number of children aged 0–5 years of age with symptoms of cough/difficulty breathing over the last two weeks.

Source: Countdown to 2015 for maternal, newborn, and child survival: the 2008 report on tracking coverage of interventions.

DHS – Demographic and Health Survey, MICS – Multiple Indicator Cluster Survey.

a In order to increase the number of observations we work with, we have included children with cough or difficulty breathing symptoms, not only pneumonia symptoms (cough + difficulty breathing).

### Coverage and equity analyses

The analysis of the indicators was two-fold: (i) for each country, coverage of the indicators was computed; and (ii) Concentration Index (CI) was used in the analysis of equity of access. CI was selected due to its numerous advantages, not least as it provides a numerical summary measure of inequity and it facilitates inter-temporal and cross-country comparisons of levels of socioeconomic-related inequality (O'Donnel et al., 2008).

The CI is a summary measure of the degree of unequal distribution of the variable of interest that places equal weights on the different degrees of inequalities along the income distribution (O'Donnel et al., 2008). It is defined “as twice the area between the concentration curve and the line of equality (the 45-degree line)” (O'Donnel et al., 2008). It can be expressed as follows (Wagstaff, 2000):

(1) $$\text{C}=\frac{2}{\mu }\left[\overset{T}{\underset{t=1}{\sum }}f_{t}\mu_{t}R_{t}\right]-1,$$

where C is the CI, $\mu =\overset{T}{\underset{t=1}{\sum }}f_{t}\mu_{t}$ expresses the overall mean quantity of the health related “good”, $\mu_{t}$ is the mean coverage rate of the *t*_*th*_ socioeconomic group, and $R_{t}$ is the relative rank of the socioeconomic group along the socioeconomic distribution of the total population. The CI is bounded between -1 and +1, where 0 reflects equality and -1 and +1 are its extreme possible values. -1 corresponds to a distribution that completely favours the poorest and +1 corresponds to a distribution that completely favours the richest. When the CI is equal to zero it can possibly mean that inequalities occurring at different points of the socioeconomic distribution have cancelled out.

The CI analysis was coupled with a decomposition analysis of the socioeconomic-related inequality affecting access. Socioeconomic-related inequality affecting a health variable of interest (captured by the CI) can be expressed as the result of the socioeconomic related inequalities of its determinants (van Doorslaer et al., 2004; Wagstaff et al., 2003).

The general model is given by equation (2) below:

(2) $$E\left(y_{i}|x_{i}\right)=G\left(\underset{k}{\sum }\beta_{k}x_{i}^{k}\right)$$

where G represents the functional form for a non-linear model. What van Doorslaer et al. proposed was to “restore the mechanics of the decomposition framework by replacing the $\beta_{k}$ parameters in equation by the $\beta_{k}^{m}$ parameters”, where the $\beta_{k}^{m}$ represent the “partial effects” of the x (the determinants of y) in the linear approximation of the non-linear model expressed by equation (3) (van Doorslaer et al., 2004):

(3) $$\;y_{i}=\underset{k}{\sum }\beta_{k}^{m}x_{i}^{k}+u_{i}$$

Consequently we conducted a decomposition analysis of the socioeconomic related inequality affecting access to selected interventions. For the decomposition analysis, the dependent variables (i.e. access to the selected key maternal and child healthcare interventions) were explained as a function of demand driven enabling factors (the mother's educational attainment, the mother's wealth index) as well as supply related proxies for community level factors (the region and the location of the residence in a rural or urban area), following the behavioural model of health service use (De La Torre et al., 2018; Wagstaff et al., 2003).

In order to draw commonalities and differences across the countries in the region, we synthesise the results by group of countries based on their HDI (human development index): (a) very high HDI countries (Qatar); (b) high HDI countries (Algeria, Jordan, Lebanon, Egypt, Tunisia); (c) medium HDI countries (Iraq, Morocco, State of Palestine); and (d) low HDI countries (Djibouti, Sudan, Syria, Yemen) (UNDP, 2019).

All analyses include the standard weights and have been conducted in Stata 14.

## Results

As the survey for the very high HDI country (Qatar) does not have information on the wealth index, we only present the results on coverage (Appendix Table A2). The coverage of selected interventions in Qatar is high (96.5% of women had four antenatal care visits, while 100% had skilled birth assistance).

The findings on coverage for selected interventions in high HDI countries are presented in Table 2. First, for the two out of three countries for which there is historical data (Egypt and Tunisia), the coverage of four antenatal care visits steadily grew during the study period. More specifically, coverage increased from 42.4% in Egypt in 2000 to 82.7% in 2014, while in the case of Tunisia, coverage of four antenatal care visits increased, albeit slightly, from 87.2% in 2012 to 88.1% in 2018. Interestingly, in the case of Jordan, the coverage of antenatal care services has been consistently high, higher than 90% and it has been teetering around the 91%–94% range. Second, for the countries for which there is historical data, the coverage of skilled delivery steadily grew over the study period. In the case of Egypt, the coverage of skilled birth assistance has gone up from 65.4% in 2000 to 91.6% in 2014, while in Jordan it has increased, albeit slightly, from 98.3% in 2002 to 99.7% in 2018. In the case of Lebanon, the coverage of skilled birth assistance was at stable 99% between 2006 and 2011, although it ought to be emphasized that the Lebanese MICS datasets cover only Palestinian refugees living in Lebanon (and hence are not representative of the entire country). Third and more importantly, over time, across the countries for which there is historical data, there has been a reduction in the share of women delivering at home. For example, the share of women delivering at home in Egypt has dropped from 47.2% in 2000 to 13.2% in 2014, while it has dropped from 3.1% in Jordan in 2002 to 0.9% in 2018. Furthermore, there is a noticeable hospital-oriented healthcare system in high HDI countries with the majority of women delivering either in public or private hospitals. Finally, most of the care seeking for diarrhoea and cough occurs in primary healthcare facilities (public and private), with a fraction occurring at secondary healthcare facilities.

**Table 2:** High HDI countries: coverage of selected maternal and child healthcare interventions (in percent).

	year	Survey	antenatal four visits	skilled delivery	delivery at home	delivery at public secondary	delivery at public primary	delivery at private secondary	delivery at private primary	diarrhoea treatment public secondary	diarrhoea treatment public primary	diarrhoea treatment private secondary	diarrhoea treatment private primary	cough treatment public secondary	cough treatment public primary	cough treatment private secondary	cough treatment private primary
Algeria	2012–13	MICS	72.6	98.3	1.4	67.5	22.2	..	8.7	..	..	..	..	20.3	30.1	9.2	31.4
Egypt	2000	DHS	42.4	65.4	47.2	23.0	1.6	28.2	..	7.1	9.2	1.7	30.3	9.8	10.0	2.9	34.4
Egypt	2005	DHS	59.2	74.6	35.3	13.3	2.0	7.7	30.7	4.7	11.1	2.0	25.9	5.5	11.0	2.7	32.7
Egypt	2008	DHS	66.5	79.0	28.2	16.0	2.1	10.5	32.8	7.4	9.4	2.5	34.5	7.3	10.3	2.7	38.1
Egypt	2014	DHS	82.7	91.6	13.2	21.2	1.4	18.8	41.7	5.1	9.3	2.4	39.8	5.6	9.7	1.6	46.2
Jordan	2002	DHS	90.9	98.3	3.1	61.8	1.7	33.3	..	5.8	26.1	4.3	18.0	7.9	33.0	5.3	20.5
Jordan	2007	DHS	94.2	99.0	1.3	61.7	0.4	33.6	..	9.6	24.4	15.4	10.2	8.1	30.2	18.6	10.3
Jordan	2012	DHS	94.5	99.6	1.2	65.2	0.1	33.5	..	10.1	27.9	13.7	8.6	7.3	36.5	14.2	9.5
Jordan	2017–18	DHS	92.0	99.7	0.9	64.0	0.5	32.3	..	9.8	14.4	7.6	15.5	7.0	19.5	6.6	18.7
Lebanon^a^	2006	MICS	..	99.4	2.1	4.1	1.1	29.1	5.0	..	..	..	..	..	..	..	..
Lebanon^a^	2011	MICS	93.9	99.2	0.7	22.6	0.5	25.1	3.9	..	..	..	..	4.8	5.1	13.1	47.6
Tunisia	2012	MICS	87.2	98.8	1.5	82.9	1.0	14.5	0.1	..	..	..	..	36.7	10.8	27.4	2.6
Tunisia	2018	MICS	88.1	99.5	0.3	75.8	1.6	22.2	0.1	25.7	4.3	7.6	31.8	24.4	8.9	3.4	26.8

Note: the coverage of delivery by place in Egypt and Jordan is influenced by the structure of the survey questions (see Supplementary material for definitions and harmonization across surveys).

DHS – Demographic and Health Survey, MICS – Multiple Indicator Cluster Survey.

a For Lebanon, the surveys are not national but they cover Palestinian refugees in Lebanon.

Table 3 presents the equity analysis results for high HDI countries. The table captures the concentration index, the standard errors as well as the statistical significance of the CI for all available surveys per country. Inter alia, this allows us to analyse the temporal dynamics in the concentration index, for countries for which there is historical data. The table lends itself to a few interesting and important findings. First, in the case of Egypt there is a decrease in the magnitude of the CI for four ante-natal care visits and skilled delivery, suggesting that, along with the increase in coverage, there has been a reduction in the pro-rich inequity in accessing this type of healthcare intervention. Second, coupling the high coverage, the CIs for selected interventions in Jordan and Tunisia (four ante-natal care visits and skilled delivery) exhibit equi-distributive patterns. For example, the CI for four ante-natal care visits in the case of Jordan has been teetering around the 0.02 mark for the last four survey waves, whilst similar findings emerge vis-à-vis skilled birth assistance in both, Jordan and Tunisia. Third, there is a split in delivery in private vs. public healthcare facilities, with delivery in private secondary facilities being consistently pro-rich, and delivery in public healthcare facilities being consistently pro-poor. Moreover, as evidenced by Table 3, this split in inequity in access has persisted over time. Finally, when considering healthcare seeking for selected childhood illnesses, for the cases where we find statistical significance of the CIs, our results show pro-poor access for care-seeking in public healthcare facilities (both, primary and secondary) and pro-rich access for cares-seeking in private healthcare facilities (primary or secondary).

**Table 3:** High HDI countries: Concentration Index for selected maternal and child health interventions.

Egypt 2000			Jordan 2002			Algeria 2012-13		
	CI	SE		CI	SE			
antenatal four visits	0.27***	0.00	antenatal four visits	0.02***	0.00		CI	SE
skilled delivery	0.17***	0.00	skilled delivery	0.01***	0.00	antenatal four visits	0.06***	0.01
delivery at home	-0.26***	0.00	delivery at home	-0.28***	0.05	skilled delivery	0.01***	0.00
delivery at public secondary	0.14***	0.01	delivery at public secondary	-0.14***	0.01	delivery at home	-0.29***	0.06
delivery at public primary	0.13***	0.03	delivery at public primary	0.1	0.06	delivery at public secondary	0	0.01
delivery at private secondary	0.28***	0.01	delivery at private secondary	0.29***	0.01	delivery at public primary	-0.07***	0.02
delivery at private primary	..	..	delivery at private primary	..	..	delivery at private secondary	..	..
diarrhoea treatment public secondary	-0.14**	0.05	diarrhoea treatment public secondary	-0.18**	0.09	delivery at private primary	0.27***	0.03
diarrhoea treatment public primary	-0.16***	0.06	diarrhoea treatment public primary	-0.21***	0.04	cough treatment public secondary	-0.1	0.06
diarrhoea treatment private secondary	0.2	0.17	diarrhoea treatment private secondary	0.18	0.16	cough treatment public primary	-0.16***	0.05
diarrhoea treatment private primary	0.16***	0.02	diarrhoea treatment private primary	0.12**	0.06	cough treatment private secondary	0.07	0.11
cough treatment public secondary	-0.20***	0.03	cough treatment public secondary	-0.14*	0.08	cough treatment private primary	0.17***	0.04
cough treatment public primary	-0.14***	0.03	cough treatment public primary	-0.15***	0.03			
cough treatment private secondary	0.24***	0.05	cough treatment private secondary	0.24*	0.12	Lebanon 2006*		
cough treatment private primary	0.17***	0.01	cough treatment private primary	0.11**	0.05		CI	SE
**Egypt 2005**			**Jordan 2007**			skilled delivery	0.00**	0.00
	CI	SE		CI	SE	delivery at home	0.07	0.12
antenatal four visits	0.19***	0.00	antenatal four visits	0.02***	0.00	delivery at public secondary	-0.25**	0.11
skilled delivery	0.12***	0.00	skilled delivery	0.00***	0.00	delivery at public primary	-0.21	0.17
delivery at home	-0.28***	0.01	delivery at home	-0.42***	0.07	delivery at private secondary	0.06**	0.03
delivery at public secondary	-0.07***	0.01	delivery at public secondary	-0.19***	0.01	delivery at private primary	0.15**	0.07
delivery at public primary	-0.07*	0.04	delivery at public primary	0.08	0.12			
delivery at private secondary	0.56***	0.02	delivery at private secondary	0.33***	0.01	Lebanon 2011*		
delivery at private primary	0.12***	0.01	delivery at private primary	..	..		CI	SE
diarrhoea treatment public secondary	-0.15**	0.05	diarrhoea treatment public secondary	-0.14**	0.06	antenatal four visits	0.01	0.01
diarrhoea treatment public primary	-0.12***	0.04	diarrhoea treatment public primary	-0.19***	0.03	skilled delivery	0	0.00
diarrhoea treatment private secondary	0.49***	0.11	diarrhoea treatment private secondary	0.26***	0.06	delivery at home	-0.82**	0.36
diarrhoea treatment private primary	0.07***	0.02	diarrhoea treatment private primary	0.18**	0.08	delivery at public secondary	0.11***	0.04
cough treatment public secondary	-0.17***	0.05	cough treatment public secondary	-0.20***	0.07	delivery at public primary	0.46	0.28
cough treatment public primary	-0.14***	0.03	cough treatment public primary	-0.17***	0.03	delivery at private secondary	0.20***	0.03
cough treatment private secondary	0.43***	0.08	cough treatment private secondary	0.31***	0.05	delivery at private primary	-0.33***	0.10
cough treatment private primary	0.06***	0.02	cough treatment private primary	0.05	0.06	cough treatment public secondary	0.21	0.31
	**Egypt 2008**		**Jordan 2012**			cough treatment public primary	-0.23***	0.13
	CI	SE		CI	SE	cough treatment private secondary	0.2	0.16
antenatal four visits	0.15***	0.00	antenatal four visits	0.02***	0.00	cough treatment private primary	0.04	0.06
skilled delivery	0.10***	0.00	skilled delivery	0.00**	0.00			
delivery at home	-0.34***	0.01	delivery at home	-0.38***	0.10	**Tunisia 2012**		
delivery at public secondary	-0.07***	0.01	delivery at public secondary	-0.15***	0.01		CI	SE
delivery at public primary	-0.14***	0.04	delivery at public primary	-0.12	0.20	antenatal four visits	0.04***	0.01
delivery at private secondary	0.51***	0.02	delivery at private secondary	0.31***	0.01	skilled delivery	0.01***	0.00
delivery at private primary	0.09***	0.01	delivery at private primary	..	..	delivery at home	-0.63***	0.15
diarrhoea treatment public secondary	-0.22***	0.06	diarrhoea treatment public secondary	-0.23***	0.06	delivery at public secondary	-0.09***	0.01
diarrhoea treatment public primary	-0.05	0.05	diarrhoea treatment public primary	-0.05	0.04	delivery at public primary	0.42	0.27
diarrhoea treatment private secondary	0.62***	0.18	diarrhoea treatment private secondary	0.20***	0.07	delivery at private secondary	0.58***	0.05
diarrhoea treatment private primary	0.02	0.03	diarrhoea treatment private primary	0.02	0.10	delivery at private primary	0.41	0.41
cough treatment public secondary	-0.21***	0.05	cough treatment public secondary	-0.16**	0.06	cough treatment public secondary	-0.09**	0.04
cough treatment public primary	-0.09**	0.04	cough treatment public primary	-0.02	0.03	cough treatment public primary	-0.11	0.11
cough treatment private secondary	0.48***	0.11	cough treatment private secondary	0.15***	0.05	cough treatment private secondary	0.32***	0.05
cough treatment private primary	0.05**	0.02	cough treatment private primary	0.07	0.06	cough treatment private primary	0.52**	0.23
**Egypt 2014**			**Jordan 2017**–**18**			**Tunisia 2018**		
	CI	SE		CI	SE		CI	SE
antenatal four visits	0.05***	0.00	antenatal four visits	0.01***	0.00	antenatal four visits	0.050***	0.01
skilled delivery	0.04***	0.00	skilled delivery	0.00***	0.00	skilled delivery	0.002**	0.00
delivery at home	-0.34***	0.01	delivery at home	-0.51***	0.09	delivery at home	-0.420*	0.25
delivery at public secondary	0.00	0.01	delivery at public secondary	-0.10***	0.01	delivery at public secondary	-0.133***	0.01
delivery at public primary	-0.13***	0.04	delivery at public primary	0.03	0.13	delivery at public primary	0.102	0.15
delivery at private secondary	0.30***	0.01	delivery at private secondary	0.23***	0.01	delivery at private secondary	0.450***	0.03
delivery at private primary	-0.04***	0.01	delivery at private primary	..	..	delivery at private primary	0.828	0.83
diarrhoea treatment public secondary	0.25***	0.06	diarrhoea treatment public secondary	-0.17**	0.08	diarrhoea treatment public secondary	-0.115	0.08
diarrhoea treatment public primary	-0.14***	0.04	diarrhoea treatment public primary	-0.11*	0.06	diarrhoea treatment public primary	-0.299	0.23
diarrhoea treatment private secondary	0.51***	0.11	diarrhoea treatment private secondary	0.24*	0.13	diarrhoea treatment private secondary	0.057	0.18
diarrhoea treatment private primary	0.00	0.02	diarrhoea treatment private primary	0.19**	0.08	diarrhoea treatment private primary	0.163**	0.07
cough treatment public secondary	0.12***	0.04	cough treatment public secondary	-0.17**	0.07	cough treatment public secondary	-0.093**	0.04
cough treatment public primary	-0.11***	0.03	cough treatment public primary	-0.11***	0.03	cough treatment public primary	-0.283***	0.08
cough treatment private secondary	0.45***	0.08	cough treatment private secondary	0.31***	0.09	cough treatment private secondary	0.382***	0.14
cough treatment private primary	0.02*	0.01	cough treatment private primary	0.10*	0.05	cough treatment private primary	0.224***	0.04

For Lebanon, the surveys are not national but they cover Palestinian refugees in Lebanon.

CI – concentration index, SE – standard errors, ***-significant at 1%, **-significant at 5%, *-significant at 10%.

Table 4 presents the synthesis of the coverage results for the medium HDI countries. A few interesting and important results emerge. First, the share of women receiving four ante-natal care visits has been increasing over time, particularly among countries that started from a lower base. For example, in Iraq, while in 2011 the coverage of four ante-natal care visits was 65.9%, it increased to 77.3% in 2018. Similar patterns for this indicator (four antenatal care visits) could be observed among the rest of the medium HDI countries for which there is more than one wave of data available. Second, the share of women delivering at home has been decreasing across all of the medium HDI countries, albeit at a different rate. For example, the share of women delivering at home in Iraq has dropped from 37.1% in 2006 to 13.3% in 2018, while around the same period, the share of women delivering at home in Morocco has decreased from 38.7% in 2003/04 to 13.8% in 2018. The share of women delivering at home in the State of Palestine has decreased from 0.8% in 2010 to 0.5% in 2014. Third, most of the deliveries occur in secondary healthcare facilities, given the hospital-oriented healthcare system. More specifically, a higher share of deliveries occur in public hospitals, compared to private ones. Finally, the coverage for care-seeking for diarrhoea and cough suggests that most of the care seeking for these common childhood illnesses occurs in primary healthcare facilities, although a fraction of the population seeks care at secondary healthcare facilities.

**Table 4:** Medium HDI countries: Coverage of selected maternal and child healthcare interventions (in percent).

		survey	antenatal four visits	skilled delivery	delivery at home	delivery at public secondary	delivery at public primary	delivery at private secondary	delivery at private primary	diarrhoea treatment public secondary	diarrhoea treatment public primary	diarrhoea treatment private secondary	diarrhoea treatment private primary	cough treatment public secondary	cough treatment public primary	cough treatment private secondary	cough treatment private primary
Iraq	2000	MICS	35.5	69.0	..	..	..	..	..	..	..	..	..	24.4	58.2	..	27.7
Iraq	2006	MICS	65.0	60.3	37.1	53.2	0.9	7.8	0.7	..	..	..	..	26.9	24.0	13.7	32.2
Iraq	2011	MICS	65.9	90.8	23.4	67.0	0.9	8.1	0.5	..	..	..	..	21.9	33.5	3.6	34.9
Iraq	2018	MICS	77.3	95.6	13.3	73.4	1.1	11.8	0.2	21.7	24.0	4.7	35.2	13.1	21.4	7.4	36.1
Morocco	2003/04	DHS	30.6	62.9	38.7	43.8	9.4	7.8	..	2.7	15.6	0.3	3.6	3.7	19.7	0.5	8.0
Morocco	2011	ENPSF	55.8	..	27.1	51.0	11.9	..	9.2	5.1	64.6	..	14.4	4.2	31.9	..	13.6
Morocco	2018	ENPSF	60.6	86.0	13.8	56.5	13.6	..	15.6	5.6	67.3	..	15.2	5.0	24.8	..	11.1
Palestine	2010	MICS	95.8	68.3	0.8	59.0	1.0	26.9	3.1	..	..	..	..	12.7	23.8	6.2	40.2
Palestine	2014	MICS	96.5	99.7	0.5	59.8	1.1	23.8	1.7	7.5	29.7	8.0	24.9	5.9	25.4	5.5	27.4

Note: in the case of Morocco 2011 and 2018, there is only information for delivery at a hospital without further disaggregation (see the Supplementary material for definitions and survey harmonization). Similarly in the case of Morocco in 2011 and 2018 there is no disaggregation by hospital type (public or private) for care-seeking for common childhood illnesses (see the Supplementary material for definitions and survey harmonization).

DHS – Demographic and Health Survey, MICS – Multiple Indicator Cluster Survey.

The equity analysis for the medium HDI countries is presented in Table 5. There are a few findings that stem from the table. First, the increase in coverage has been coupled with a reduction in pro-rich inequity in access to selected interventions. For example, the CI for four antenatal care visits in the case of Morocco has reduced from 0.31 in 2003–04 to 0.10 in 2018. Second, similarly to the high HDI countries, interventions with coverage above 90% show a consistent equi-distributive patterns, as the CIs for skilled delivery in the case of Iraq (2011 and 2018) show. Third, we find a persistent split in delivery at public vs. private secondary healthcare facilities, with pro-rich inequity in delivery at private and pro-poor inequity in delivery at public secondary healthcare facilities. Fourth, the results (for which there is statistical significance in the CI) show pro-poor inequity in access for care-seeking for diarrhoea and cough in the public sector (both, primary and secondary healthcare facilities) and pro-rich inequity in access for care-seeking in the private sector.

**Table 5:** Medium HDI countries: Concentration Index for selected maternal and child healthcare interventions.

Iraq 2000			Morocco 2011		
				CI	SE
	CI	SE	antenatal four visits	0.15***	0.01
antenatal four visits	0.07***	0.02	skilled delivery	..	..
skilled delivery	0.10***	0.01	delivery at home	-0.41***	0.01
cough treatment public secondary	0.07	0.05	delivery at public secondary	0.12***	0.01
cough treatment public primary	-0.01	0.02	delivery at public primary	-0.04**	0.02
cough treatment private secondary	..	..	delivery at private secondary	..	..
cough treatment private primary	0.01	0.04	delivery at private primary	0.62***	0.03
			diarrhoea treatment public secondary	0.06	0.16
**Iraq 2011**			diarrhoea treatment public primary	-0.04	0.03
			diarrhoea treatment private secondary	..	..
	CI	SE	diarrhoea treatment private primary	0.34***	0.10
antenatal four visits	0.04***	0.01	cough treatment public secondary	0.16**	0.06
skilled delivery	0.03***	0.00	cough treatment public primary	-0.12***	0.02
delivery at home	-0.14***	0.01	cough treatment private secondary	..	..
delivery at public secondary	0.00	0.01	cough treatment private primary	0.27***	0.04
delivery at public primary	-0.17***	0.06	**Morocco 2018**		
delivery at private secondary	0.39***	0.03		CI	SE
delivery at private primary	-0.09	0.09	antenatal four visits	0.10***	0.01
cough treatment public secondary	-0.02	0.03	skilled delivery	0.07***	0.00
cough treatment public primary	-0.13***	0.02	delivery at home	-0.43***	0.02
cough treatment private secondary	0.07	0.08	delivery at public secondary	0.04***	0.01
cough treatment private primary	0.15***	0.02	delivery at public primary	-0.17***	0.02
			delivery at private secondary	..	..
**Iraq 2018**			delivery at private primary	0.38***	0.02
			diarrhoea treatment public secondary	0.05	0.15
	CI	SE	diarrhoea treatment public primary	-0.02	0.02
antenatal four visits	0.04***	0.01	diarrhoea treatment private secondary	..	..
skilled delivery	0.01***	0.00	diarrhoea treatment private primary	0.15*	0.08
delivery at home	-0.11**	0.05	cough treatment public secondary	0.13*	0.07
delivery at public secondary	-0.04***	0.01	cough treatment public primary	-0.11***	0.03
delivery at public primary	0.01	0.07	cough treatment private secondary	..	..
delivery at private secondary	0.37***	0.05	cough treatment private primary	0.18***	0.04
delivery at private primary	-0.64	0.56	**State of Palestine 2010**		
				CI	SE
diarrhoea treatment public secondary	0.18**	0.07	antenatal four visits	0.01***	0.00
diarrhoea treatment public primary	-0.09**	0.04	skilled delivery	-0.01**	0.01
diarrhoea treatment private secondary	-0.10	0.12	delivery at home	-0.25**	0.11
diarrhoea treatment private primary	0.05	0.04	delivery at public secondary	-0.10***	0.01
cough treatment public secondary	0.01	0.05	delivery at public primary	0.07	0.10
cough treatment public primary	0.01	0.04	delivery at private secondary	0.20***	0.01
cough treatment private secondary	-0.02	0.07	delivery at private primary	-0.10**	0.05
cough treatment private primary	0.08**	0.03	cough treatment public secondary	-0.16**	0.07
			cough treatment public primary	-0.12**	0.05
**Morocco 2003**–**04**			cough treatment private secondary	0.11	0.10
			cough treatment private primary	0.19***	0.03
	CI	SE	**State of Palestine 2014**		
antenatal four visits	0.31***	0.01		CI	SE
skilled delivery	0.22***	0.00	antenatal four visits	0	0.00
delivery at home	-0.34***	0.01	skilled delivery	0.01	0.00
delivery at public secondary	0.20***	0.01	delivery at home	-0.09	0.13
delivery at public primary	-0.11***	0.02	delivery at public secondary	-0.17***	0.01
delivery at private secondary	0.70***	0.03	delivery at public primary	-0.26**	0.11
delivery at private primary	..	..	delivery at private secondary	0.27***	0.02
			delivery at private primary	-0.39***	0.09
diarrhoea treatment public secondary	0.30**	0.15	diarrhoea treatment public secondary	-0.16**	0.08
diarrhoea treatment public primary	0.02	0.05	diarrhoea treatment public primary	-0.22***	0.04
diarrhoea treatment private secondary	0.23	0.41	diarrhoea treatment private secondary	0.39***	0.08
diarrhoea treatment private primary	0.49***	0.14	diarrhoea treatment private primary	0.34***	0.04
cough treatment public secondary	0.19***	0.07	cough treatment public secondary	-0.24***	0.05
cough treatment public primary	0.01	0.02	cough treatment public primary	-0.09***	0.02
cough treatment private secondary	0.59***	0.21	cough treatment private secondary	0.29***	0.06
cough treatment private primary	0.47***	0.05	cough treatment private primary	0.29***	0.02

CI – concentration index, SE – standard errors, ***-significant at 1%, **-significant at 5%, * - significant at 10%.

The final set of results synthesise the findings on coverage and equity for selected and available low HDI countries. Table 6 summarizes the results on coverage for selected interventions. First, the coverage of some of the indicators (e.g. four ante-natal care visits) is lower compared to the other groups of countries although it shows some improvement over time. Second, for the countries for which there is more than one survey wave, it is encouraging that the share of women whose delivery is attended by a skilled staff has been increasing. Third, in the cases of Yemen and Sudan, a large share of women continue to deliver at home (e.g. 71.9% in Sudan in 2014), although, as the case of Yemen shows, the share of women delivering at home has been decreasing. The rest of the deliveries occur in public hospitals. Finally, most of the care-seeking for diarrhoea and cough occurs in the public primary healthcare facilities.

**Table 6:** Low HDI countries: coverage of selected maternal and child healthcare interventions (in percent).

		survey	antenatal four visits	skilled delivery	delivery at home	delivery at public secondary	delivery at public primary	delivery at private secondary	delivery at private primary	diarrhoea treatment public secondary	diarrhoea treatment public primary	diarrhoea treatment private secondary	diarrhoea treatment private primary	cough treatment public secondary	cough treatment public primary	cough treatment private secondary	cough treatment private primary
Djibouti	2006	MICS	..	92.9	5.6	81.5	0.8	6.0	0.2	..	..	..	..	49.1	17.5	12.1	4.7
Sudan	2010	MICS	62.3	74.1	..	..	..	..	..	24.7	47.3	2.4	6.0	22.6	51.7	3.2	8.4
Sudan	2014	MICS	64.1	77.2	71.9	25.5	0.9	1.5	0.1	25.5	37.5	5.4	3.7	25.4	39.9	4.4	4.3
Syria	2006	MICS	..	92.2	29.2	29.3	1.5	28.8	10.6	..	..	..	..	10.1	19.9	8.8	57.1
Yemen	2006	MICS	..	35.8	75.6	15.8	0.7	4.7	1.9	..	..	..	..	..	..	..	..
Yemen	2013	DHS	25.3	45.3	69.3	17.2	2.0	10.2	..	8.9	10.6	13.7	..	7.8	9.8	13.3	..

DHS – Demographic and Health Survey, MICS – Multiple Indicator Cluster Survey.

Table 7 summarizes the results on the equity analysis for some of the main indicators for low HDI countries. First, the equity analysis shows that there is a pronounced pro-rich inequity in access to four antenatal care visits. Second, the CI for skilled birth assistance is larger than 0.1 suggesting a more pronounced pro-rich inequity. For example, the CI for skilled delivery in the case of Sudan in 2014 was 0.13, while it was similarly high (0.27) in the case of Yemen 2013. Third, there is a consistent pro-rich inequity in delivery in any healthcare facility, regardless if it's public or private. The results also suggest a pro-rich inequity in access to selected child care interventions (in the cases where we find statistical significance for the CIs). The only exception is Yemen 2013, where the sign of the CI indicates pro-poor inequity in access to care-seeking for diarrhoea and cough at primary public healthcare facilities.

**Table 7:** Low HDI countries: Concentration Index for selected maternal and child healthcare interventions.

Sudan 2010			Syria 2006		
				CI	SE
	CI	SE	skilled delivery	0.04***	0.00
antenatal four visits	0.10***	0.01	delivery at home	-0.15***	0.01
skilled delivery	0.12***	0.01	delivery at public secondary	-0.10***	0.01
			delivery at public primary	0.00	0.07
diarrhoea treatment public secondary	0.10***	0.03	delivery at private secondary	0.28***	0.01
diarrhoea treatment public primary	0.00	0.02	delivery at private primary	-0.07***	0.03
diarrhoea treatment private secondary	0.38***	0.13	cough treatment public secondary	0.02	0.08
diarrhoea treatment private primary	0.19**	0.07	cough treatment public primary	0.00	0.05
cough treatment public secondary	0.10***	0.03	cough treatment private secondary	0.06	0.07
cough treatment public primary	-0.03*	0.01	cough treatment private primary	0.06**	0.02
cough treatment private secondary	0.38***	0.09	**Yemen 2006**		
cough treatment private primary	0.24***	0.05		CI	SE
			skilled delivery	0.30***	0.02
**Sudan 2014**			delivery at home	-0.11***	0.01
			delivery at public secondary	0.38***	0.04
	CI	SE	delivery at public primary	0.02	0.19
antenatal four visits	0.09***	0.01	delivery at private secondary	0.27***	0.08
skilled delivery	0.13***	0.00	delivery at private primary	0.24**	0.11
delivery at home	-0.15***	0.01	**Yemen 2013**		
delivery at public secondary	0.37***	0.02		CI	SE
delivery at public primary	0.12	0.13	antenatal four visits	0.37***	0.01
delivery at private secondary	0.72***	0.12	skilled delivery	0.27***	0.01
delivery at private primary	0.81**	0.36	delivery at home	-0.13***	0.00
			delivery at public secondary	0.32***	0.01
diarrhoea treatment public secondary	-0.01	0.02	delivery at public primary	0	0.04
diarrhoea treatment public primary	0.08***	0.02	delivery at private secondary	0.30***	0.02
diarrhoea treatment private secondary	0.14*	0.07	delivery at private primary	..	..
diarrhoea treatment private primary	0.10	0.09	diarrhoea treatment public secondary	0.129***	0.03
cough treatment public secondary	0.05**	0.02	diarrhoea treatment public primary	-0.158***	0.03
cough treatment public primary	0.03*	0.02	diarrhoea treatment private secondary	0.181***	0.03
cough treatment private secondary	0.24***	0.07	diarrhoea treatment private primary	..	..
cough treatment private primary	0.17**	0.08	cough treatment public secondary	0.117***	0.03
			cough treatment public primary	-0.118***	0.03
			cough treatment private secondary	0.191***	0.03
			cough treatment private primary	..	..

CI – concentration index, SE – standard errors, ***-significant at 1%, **-significant at 5%, *-significant at 10%.

The Appendix Tables (A3-A26) provide a snapshot of the decomposition analysis for the selected interventions with a pro-rich inequity in access. There are two main results that stem from this analysis. First, the demand side enabling factors (socio-economic status, i.e. wealth as well as the education of the woman/mother are the main drivers of the pro-rich inequity in access to selected interventions). Second, we also find evidence for the importance of the supply side factors, which, in part, explain the pro-rich inequity of access in selected interventions. More specifically, we find that urbanicity (i.e. the urban/rural divide) contributes to higher pro-rich inequity in selected interventions. This finding could be a direct result of the fact that hospitals, and in particular, private hospitals are placed in urban areas, thus further contributing to the pro-rich inequity in access to these facilities. It is worth pointing out that these patterns established through the decomposition analysis hold for the various types of countries included in the analysis.

## Discussion

There are several important findings that emerge from our analysis. First, in countries that started from lower base there has been an increase in the coverage of selected interventions delivered in PHC setting (e.g. four ante-natal care visits) coupled with an increase in equity of access. On the other hand, countries with consistently high coverage of selected interventions (i.e. coverage over 90%) have shown a consistent equi-distributive pattern in access to selected healthcare interventions. Second, over time and in countries that have started from a lower base, there has been a reduction in share of women delivering at home coupled with an increase in the coverage and equity of skilled birth assistance. By contrast, countries with high institutional delivery have exhibited an equi-distributive pattern in the skilled birth assistance during the study period. Third, except in the low HDI countries, in the rest of the MENA region, there is clear split indicating pro-rich inequity in delivery at private secondary healthcare facilities and pro-poor inequity in delivery at public secondary healthcare facilities. In other words, there is a de-facto two-tiered system, where rich gravitate towards the private sector, and the poor towards the public sector. Fourth, while the highest share of care-seeking for common child illnesses (such as diarrhoea and cough) occurs in primary healthcare facilities, there is a fraction of care (about 20–30 percent) for childhood illnesses that takes place in secondary healthcare facilities. More specifically, the equity analysis, in the cases where we find statistical significance for the CIs, suggests that households in the richer quintiles are more likely to seek care for the common child illnesses in private healthcare facilities (primary and secondary) while those in the poorer wealth quintiles are more likely to go in public healthcare facilities (primary and secondary).

Our findings suggest that in the last two decades, in high and medium HDI countries that started from a lower base, PHC has been the key contributor in increasing the coverage and in reducing the rich-poor gap, particularly in relation to maternal healthcare interventions. This, in part could be due to some of the initiatives that have been introduced in selected countries. In Egypt, for example, the Health Sector Reform Program (HSRP) initiated in 1997 was designed to change the care delivery approach for PHC facilities through the creation of the family health model and with an objective to meet the needs of the population through a responsive and comprehensive package of services that included maternal and child health services, family planning, immunizations and management of childhood illnesses. More specifically, family doctors at each PHC unit acted as gatekeepers for specialty services, to decrease the burden on secondary care and better integrate service provision at the facility level. In addition, the programme also prioritized improving and ensuring quality of care by, inter alia: providing formal, specialized family health training, implementation of standards for accreditation of PHC facilities using the family physician model, performance-based incentives for clinicians as well as improvements in facility infrastructure (Al Bahnasy et al., 2016; El Rabbat & Bossert, 2009).

However, our results for both, high and medium HDI countries suggest that as the coverage of selected interventions increases above 90% (e.g. antenatal care, skilled birth assistance in the case of Jordan), further progress has been occurring at a slower rate (or stalling altogether), as the case of Jordan goes on to show. While this is a reflection of the law of diminishing returns, it could partly illustrate the ‘hardest mile’ argument – that achieving an improvement in coverage from 50% to 70% might be less difficult and costly than achieving a coverage from 90% to 95%. In other words, the process is not linear, particularly when it comes to reaching those that are hardest to reach (e.g. ultra-poor, rural, etc.) (Nikoloski & Mossialos, 2018).

Furthermore, when considering the maternal healthcare interventions, we find evidence for a rich-poor, public-private split in that there is pro-rich access to private healthcare facilities and pro-poor access to public healthcare facilities. These utilization patterns are a direct result of the public-private segmentation of healthcare delivery in the wider MENA region as well as the socio-economic disparities between the poor and the rich (World Health Organization, 2015). There are some examples, however, particularly in countries with limited fiscal space, where this split has worked in favour of increasing coverage of selected interventions, by encouraging the public and private sectors to work together and share the burden of provision of PHC services. In Lebanon, in an attempt to increase the accessibility of PHC services, the Ministry of Public Health developed a special type of contractual agreement with public and private centres (including NGOs) that fit a delineated set of criteria. This has led to the creation and expansion of Lebanon's National PHC Network distributed across Lebanon's eight provinces. In addition, this also allowed for PHC centres to be distributed according to catchment areas, where each area has an average of 15,000–20,000 inhabitants (Ministry of Public Health, 2017).

Our results suggest that some care-seeking for childhood illnesses that could be equally handled at primary level occurs at secondary level. In addition, in the cases where the CIs are statistically significant, we find evidence for a pro-rich access to care-seeking in the private sector (both primary and secondary) and pro-poor access in the public sector. While previous research has only analysed the public/private split (Chakraborty & Sprockett, 2018), our analysis goes a step further suggesting a limited impact of the ‘PHC approach’ in decongesting secondary health facilities. The findings are a reflection of the hospital-centred healthcare systems in the region, with lack of focus on preventative care (Agreus & Saab, 2015). Moreover, our results also suggest that while people could be more comfortable going to PHC for ‘preventive’ interventions like immunization (UNICEF, 2020), they gravitate towards hospitals for interventions like treatment for sick children, as they perceive secondary healthcare to be of superior quality (Alami et al., 2015).

We also find significant heterogeneity across country groups with low HDI countries lagging behind in both, coverage and equity of access. While most of the maternal services in these countries are free of charge (Mustafa & Mukhtar, 2015), this finding suggests that there are additional barriers to seeking care (e.g. transport, cost for accompanied person) or that other factors, such as perception of quality of healthcare, play a role in the overall access to healthcare (Kabakian-Khasholian et al., 2000). Moreover, in the low HDI countries, our results attest that even the publicly-funded health services are used more by the rich than the poor, suggesting that government health spending benefits the rich more than the poor. Therefore, the low HDI countries are similar to what has previously been established in a Sub-Saharan African context (Castro-Leal et al., 2000; Zere et al., 2012).

Finally, the decomposition analysis confirms the findings from the literature review that certain socio-economic enabling factors (e.g. wealth or maternal education) contribute to the pro-rich inequity in the selected intervention. Moreover, the results also point to the fact that supply side factor (proxied by residence (urban vs. rural)) positively contribute to the pro-rich inequity in access to the selected services (Couillet et al., 2009; Obermeyer & Potter, 1991; Siziya et al., 2009).

There are some limitations associated with this study. Fist, the analysis was conducted with cross-sectional data that do not allow causal interpretation of the findings. Second, whilst the MICS and DHS surveys are comparable across countries and over time, there are some differences in the way certain questions were worded, although, to the best of our knowledge we have harmonized each variable as much as possible. Third, the most recent available surveys for some of the countries, currently going through conflict, are relatively outdated (e.g. Syria, Yemen) and this has obvious limitations on the validity of the findings for these countries. Finally, availability of survey data for the Gulf countries (as well as survey variables for the wealth index) prevented us from including most of them in the equity analysis.

## Conclusion and policy implications

This study has important policy implications. In line with the renewed focus on PHC following the Astana declaration, countries across the region should invest more in strengthening and expansion of the PHC network, with further focus on improving quality of care and community engagement. These improvements could also help reorient the healthcare systems in the region from their current and heavy focus on hospital care to a more cost-effective hybrid model that includes a strong PHC component providing relevant services. In particular, expanding the network of primary healthcare centres in peri-urban and rural areas and further investment in community healthcare workers with strengthening of the referral system could significantly improve coverage and equity of interventions, for maternal and child health. Strengthening of the referral system and effective engagement with communities would also promote timely seeking of healthcare for interventions that could be cheaply delivered at PHC setting (e.g. care seeking for common childhood illness such as diarrhoea and cough). Given the limited fiscal space, there should be better coordination of the publicly and privately provided healthcare including through an effective engagement with the private health sector. Specifically, countries need to develop policy frameworks, organizational systems and financing strategies that facilitate the role of the private sector in health service delivery while ensuring quality of services. Finally, all of these policy interventions become even more significant in the context of the COVID-19 pandemic as PHC is the first point of contact for the population's health needs and also serves as a means for restoring the communities' trust in the health system when fear of contracting the virus decreases utilization of health services across the region.

## Author contribution

Zlatko Nikoloski, Hrayr Wannis and Anirban Chatterjee designed the study. Zlatko Nikoloski conducted the data analyses with inputs from Hrayr Wannis and Leonardo Menchini. All authors drafted the final version of the manuscript.
