# Supplementary material for: Primary healthcare and child and maternal health in the Middle East and North Africa (MENA): A retrospective analysis of 29 national survey data from 13 countries
Source: SSM Popul Health. 2021 Jan 12;13:100727. doi: 10.1016/j.ssmph.2021.100727 (PMC7823042; doi:10.1016/j.ssmph.2021.100727)
Supplement: Multimedia component 1 [file mmc1.docx]

***Supplementary material***

*Definitions*

Skilled delivery, as per UNICEF/WHO definition is defined to include delivery by a doctor, nurse or a midwife. All other help (e.g. community health worker, friend etc) are grouped under unskilled delivery.

When it comes to place of delivery, we have created five main categories: delivery at home, delivery in a public secondary healthcare (e.g. government hospital, university hospital), public primary healthcare (e.g. healthcare clinic, government healthcare centre), private secondary healthcare (e.g. private hospital), and private primary healthcare (e.g. private healthcare clinic, private MHC clinic etc). All other places of delivery (e.g. NGO healthcare centres, other) are considered as other. Some surveys required further harmonization, elaborated below.

Similarly to above, we have created four groups of places for seeking care for diarrhoea and cough symptoms: public secondary, public primary, private secondary, private primary. Some surveys required further harmonization, elaborated below.

*Harmonization of surveys across countries and definitions of different categories*

Algeria

- The module in Algeria does not have an option for not receiving ante-natal care;
- The Algerian module does not have an option of private hospital when it comes to a place of delivery;
- There is however an option of delivering the baby in CHU (centre hospital universitaire). This was considered as a secondary public healthcare.
- When it comes to the care seeking for cough symptoms (for children), primary public healthcare is defined to include: ‘centre de sante du gouvernement’ and ‘poste de sante du gouvernment’;
- When it comes to the care seeking for cough symptoms (for children), primary private healthcare is defined to include: medcin prive;

Djibouti

- In the case of Djibouti, there is no wealth index, so the standard concentration analysis couldn’t be performed;
- In the case of Djibouti, in the case of delivery at birth, there is an option of ‘sage femme auxiliaire’; as this is a form of a midwife, this was grouped as skilled delivery;
- When it comes to the care seeking for cough symptoms (for children), primary public healthcare is defined to include: 'centre de sante gouvernementale', 'poste de sante gouvernmentale', 'agent de sante villageoise', 'clinique mobile/communautaire';
- When it comes to the care seeking for cough symptoms (for children), primary private healthcare is defined to include: medecin prive', and 'clinique mobile privee’;

Egypt

- in the DHS for 2000, the private hospital and clinic are grouped under one category. This was considered as a ‘private secondary care’;
- In the Egyptian case in the DHS for 2005, 2008 and 2014 for place of delivery in the private sector, there are two options: private hospital/clinic (this was treated as private secondary) and private doctor (this was treated as private primary);
- In the case of care seeking for diarrhoea and cough, primary public healthcare in this case is defined to include 'PHCU', 'health office' and 'FHU'; the primary private healthcare only includes private doctor (the only option available for primary private healthcare);

Iraq

- When it comes to the ‘place of delivery’ Iraq 2018 MICS also has an option of the house of the ‘midwife’. This option is still included as ‘delivery at home’;
- The 2006 MICS on the ante-natal care question has an option of ‘licenced’ and ‘not licensed’ care. These were grouped under ‘other’;
- The 2000 MICS when it comes to the question of delivery, it doesn’t have options on public vs. private;
- In Iraq in 2018, when it comes to care seeking for diarrhoea and cough, primary public healthcare is defined to include: 'government health centre', ' healthy home', ' government health post', 'mobile/outreach clinic'; the private primary healthcare includes: primary private physician or primary medical assistant; Same applies for the earlier years as well. In the 2000 wave, the options are somewhat different and primary public healthcare in this case is defined to include: health centre, dispensary, village health worker, MCH clinic, mobile/outreach clinic;

Jordan

- Same as in the case of Egypt above on the DHS questions in that private hospital and private primary clinic appear under one and the same category;
- In the case of Jordan in DHS 2007, in the case of place of delivery, there is an option of ‘Royal Medical Services’. This was grouped under ‘public secondary’. Same goes for the Jordan 2012 DHS and Jordan 2017/2018 DHS data;

Lebanon

- When it comes to the place of delivery, in the case of the Palestinian refugees in Lebanon 2011, the following categories were excluded: RPC hospital and UNRWA clinic as they both fall outside of the usual public/private division of healthcare; Same for the RPC option in the 2006 case;
- When it comes to healthcare seeking for diarrhoea and cough symptoms, based on the 2011 dataset, public primary healthcare is defined to include: government health centre, government health post, village health worker, mobile/outreach clinic;

Morocco

- When it comes to the DHS dataset (2003) same issues on the place of delivery apply here as well in that private hospital and private doctor appear under one category. They are hence treated as ‘private secondary’.
- In the 2011 dataset, when it comes to place of delivery, the following categories were combined: ‘clinique privee’ and ‘medcin prive’ under ‘private primary’;
- In the 2011 dataset, when it comes to healthcare seeking for diarrhoea and cough symptoms, public primary healthcare is defined to include: entre de sante and equipe mobile;
- The 2011 and 2018 datasets only have questions on any hospital (without specifying if it’s public or private). For the purpose of this research, it is considered as ‘public secondary facility’ though with the obvious caveat attached to it.

State of Palestine

- In the case of Palestine, when it comes to place of delivery, NGO hospitals, UNRWA hospitals as well as Israeli hospitals are not counted towards the public or the private sector. This applies to both datasets for 2010 and for 2014;
- Same applies to the healthcare seeking for child health (NGO hospitals, Israeli hospitals, UNRWA hospitals) are excluded from the analysis;

Qatar

- Nothing in addition;

Sudan

- In the case of Sudan 2010, the skilled delivery at birth includes: doctor, nurse/midwife or village midwife;
- The place of delivery does not have a disaggregation on private/public, so we could only present the bulky results, i.e. delivery at a hospital, etc.

Syria

- No further issues in addition to the definitions at the beginning of this methodological note;

Tunisia

- No further issues in addition to the definitions at the beginning of this methodological note;

Yemen

- In the case of the DHS 2013 dataset, the same issues as the one raised under the Egyptian case apply here as well in that private hospital and private doctor appear under one category;

Appendix Tables

**Table A1. Available surveys, sample composition and modules**

| **country** | **Survey** | **total sample - women** | **total sample - children below 5** | **questions on ante-natal care** | **questions on place of delivery** | **questions on assisted delivery** | **questions on care seeking for child illnesses** |
| --- | --- | --- | --- | --- | --- | --- | --- |
| Algeria | Algeria MICS 2012-13 | 38548 | 15140 | √ | √ | √ | √ |
| Djibouti | Djibouti MICS 2006 | 6020 | 2245 | √ | √ | √ | √ |
| Iraq | Iraq MICS 2000 | 23000 | 14676 | √ | √ | √ | √ |
| Iraq | Iraq MICS 2006 | 27186 | 16469 | √ | √ | √ | √ |
| Iraq | Iraq MICS 2011 | 55194 | 36307 | √ | √ | √ | √ |
| Iraq | Iraq MICS 2018 | 30,660 | 16,623 | √ | √ | √ | √ |
| Lebanon | Lebanon MICS 2006 | 3955 | 2381 | √ | √ | √ | √ |
| Lebanon | Lebanon MICS 2011 | 5568 | 1922 | √ | √ | √ | √ |
| Morocco | Morocco MICS 2011 | 11,069 | 11,069 | √ | √ | √ | √ |
| Morocco | Morocco ENPSF 2018 | 9,969 | 9,969 | √ | √ | √ | √ |
| Palestine, State of | State of Palestine MICS 2010 | 11173 | 7900 | √ | √ | √ | √ |
| Palestine, State of | State of Palestine MICS 2014 | 13367 | 7816 | √ | √ | √ | √ |
| Qatar | Qatar MICS 2012 | 5701 | 2082 | √ | √ | √ | √ |
| Sudan | Sudan MICS 2010 | 17174 | 13282 | √ | √ | √ | √ |
| Sudan | Sudan MICS 2014 | 18302 | 14081 | √ | √ | √ | √ |
| Syria | Syria MICS 2006 | 25563 | 11017 | √ | √ | √ | √ |
| Tunisia | Tunisia MICS 2011-12 | 10215 | 2899 | √ | √ | √ | √ |
| Tunisia | Tunisia MICS 2018 | 10,559 | 3420 | √ | √ | √ | √ |
| Yemen | Yemen MICS 2006 | 3742 | 3783 | √ | √ | √ | √ |
| Egypt | DHS 2000 | 15573 | 11361 | √ | √ | √ | √ |
| Egypt | DHS 2005 | 19474 | 13600 | √ | √ | √ | √ |
| Egypt | DHS 2008 | 16527 | 10590 | √ | √ | √ | √ |
| Egypt | DHS 2014 | 21762 | 11391 | √ | √ | √ | √ |
| Jordan | DHS 2002 | 6006 | 5820 | √ | √ | √ | √ |
| Jordan | DHS 2007 | 10876 | 9421 | √ | √ | √ | √ |
| Jordan | DHS 2012 | 11352 | 9833 | √ | √ | √ | √ |
| Jordan | DHS 2017-18 | 24402 | 9454 | √ | √ | √ | √ |
| Morocco | DHS 2003-04 | 16798 | 4695 | √ | √ | √ | √ |
| Yemen | DHS 2013 | 16656 | 4655 | √ | √ | √ | √ |

**Table A2. Very high HDI countries (Qatar): coverage of selected indicators (in percent)**

| **Indicator** | | **Country** | **year** | **overall coverage (%)** |
| --- | --- | --- | --- | --- |
|  |  |  |  |  |
|  |  |  |  |  |
|  |  |  |  |  |
| antenatal four visits | | Qatar | 2012 | 96.5 |
| place of delivery | home | Qatar | 2012 | 0.1 |
|  | public secondary | Qatar | 2012 | 83.6 |
|  | public primary | Qatar | 2012 | 1.5 |
|  | private secondary | Qatar | 2012 | 12.4 |
|  | private primary | Qatar | 2012 | 1.2 |
| skilled delivery | | Qatar | 2012 | 100 |
| Care seeking for diarrhoea | public secondary | Qatar | 2012 |  |
|  | public primary | Qatar | 2012 |  |
|  | private secondary | Qatar | 2012 |  |
|  | private primary | Qatar | 2012 |  |
| Care seeking for cough | public secondary | Qatar | 2012 | 71.63 |
|  | public primary | Qatar | 2012 | 64.27 |
|  | private secondary | Qatar | 2012 | 3.4 |
|  | private primary | Qatar | 2012 | 2.4 |

**Appendix table A3. Algeria 2012-2013. Decomposition of CI for selected pro-rich interventions**

|  | Algeria 2012-13 | | | | | |
| --- | --- | --- | --- | --- | --- | --- |
|  |  | elasticities | CI | contribution to CI | contribution to CI (%) | p values |
| skilled delivery | urban | 0.003 | 0.217 | 0.001 | 0.125 | 0.090 |
|  | education | 0.007 | 0.114 | 0.001 | 0.142 | 0.001 |
|  | wealth | 0.010 | 0.265 | 0.003 | 0.440 | 0.004 |
|  | region | -0.012 | -0.013 | 0.000 | 0.026 | 0.000 |
| cough treatment private secondary | urban | 0.265 | 0.212 | 0.056 | 0.867 | 0.272 |
|  | education | 0.201 | 0.142 | 0.029 | 0.439 | 0.539 |
|  | wealth | 0.048 | 0.275 | 0.013 | 0.203 | 0.920 |
|  | region | -0.040 | -0.015 | 0.001 | 0.009 | 0.868 |
|  |  |  |  |  |  |  |
| cough treatment private primary | urban | 0.074 | 0.212 | 0.016 | 0.095 | 0.464 |
|  | education | 0.166 | 0.143 | 0.024 | 0.144 | 0.252 |
|  | wealth | 0.516 | 0.275 | 0.142 | 0.860 | 0.005 |
|  | region | -0.069 | -0.013 | 0.001 | 0.005 | 0.499 |

**Appendix table A4. Egypt 2000. Decomposition of CI for selected pro-rich interventions**

| Egypt 2000 | | | | | | |
| --- | --- | --- | --- | --- | --- | --- |
|  |  | elasticities | CI | contribution to CI | contribution to CI (%) | p values |
| skilled delivery | urban | 0.042 | 0.255 | 0.011 | 0.064 | 0.000 |
|  | education | 0.118 | 0.209 | 0.025 | 0.144 | 0.000 |
|  | wealth | 0.424 | 0.215 | 0.091 | 0.536 | 0.000 |
|  | region | -0.099 | -0.100 | 0.010 | 0.058 | 0.000 |
| delivery at public secondary | urban | 0.241 | 0.255 | 0.061 | 0.441 | 0.000 |
|  | education | -0.081 | 0.209 | -0.017 | -0.122 | 0.000 |
|  | wealth | 0.229 | 0.215 | 0.049 | 0.354 | 0.000 |
|  | region | -0.130 | -0.100 | 0.013 | 0.093 | 0.000 |
| delivery at public primary | urban | 0.134 | 0.256 | 0.034 | 0.272 | 0.041 |
|  | education | -0.045 | 0.209 | -0.009 | -0.074 | 0.472 |
|  | wealth | 0.047 | 0.215 | 0.010 | 0.080 | 0.836 |
|  | region | -0.400 | -0.099 | 0.040 | 0.315 | 0.001 |
| delivery at private secondary | urban | -0.137 | 0.255 | -0.035 | -0.123 | 0.000 |
|  | education | 0.305 | 0.208 | 0.064 | 0.224 | 0.000 |
|  | wealth | 0.782 | 0.215 | 0.168 | 0.592 | 0.000 |
|  | region | -0.256 | -0.101 | 0.026 | 0.091 | 0.000 |
| diarrhoea treatment private secondary | urban | -0.256 | 0.256 | -0.066 | -0.321 | 0.242 |
|  | education | -0.270 | 0.210 | -0.057 | -0.278 | 0.024 |
|  | wealth | 1.486 | 0.215 | 0.319 | 1.566 | 0.015 |
|  | region | 0.273 | -0.101 | -0.028 | -0.136 | 0.415 |
| cough treatment private secondary | urban | 0.323 | 0.256 | 0.083 | 0.340 | 0.009 |
|  | education | -0.073 | 0.209 | -0.015 | -0.063 | 0.515 |
|  | wealth | 0.674 | 0.215 | 0.145 | 0.596 | 0.079 |
|  | region | 0.123 | -0.100 | -0.012 | -0.050 | 0.497 |

**Appendix table A5. Egypt 2005. Decomposition of CI for selected pro-rich interventions**

| Egypt 2005 | | | | | | |
| --- | --- | --- | --- | --- | --- | --- |
|  |  | elasticities | CI | contribution to CI | contribution to CI (%) | p values |
| skilled delivery | urban | 0.035 | 0.411 | 0.014 | 0.116 | 0.000 |
|  | education | 0.102 | 0.333 | 0.034 | 0.277 | 0.000 |
|  | wealth | 0.235 | 0.278 | 0.065 | 0.535 | 0.000 |
|  | region | -0.129 | -0.153 | 0.020 | 0.162 | 0.000 |
| delivery at private secondary | urban | 0.096 | 0.411 | 0.039 | 0.070 | 0.000 |
|  | education | 0.210 | 0.333 | 0.070 | 0.124 | 0.000 |
|  | wealth | 0.982 | 0.278 | 0.273 | 0.487 | 0.000 |
|  | region | -0.253 | -0.153 | 0.039 | 0.069 | 0.000 |
| delivery at private primary | urban | -0.102 | 0.411 | -0.042 | -0.356 | 0.000 |
|  | education | 0.186 | 0.333 | 0.062 | 0.524 | 0.000 |
|  | wealth | 0.325 | 0.278 | 0.090 | 0.766 | 0.000 |
|  | region | -0.057 | -0.153 | 0.009 | 0.073 | 0.000 |
| diarrhoea treatment private secondary | urban | 0.057 | 0.411 | 0.024 | 0.048 | 0.657 |
|  | education | -0.214 | 0.332 | -0.071 | -0.145 | 0.120 |
|  | wealth | 1.168 | 0.278 | 0.325 | 0.665 | 0.003 |
|  | region | -0.539 | -0.154 | 0.083 | 0.170 | 0.035 |
| cough treatment private secondary | urban | 0.049 | 0.410 | 0.020 | 0.047 | 0.602 |
|  | education | -0.068 | 0.332 | -0.023 | -0.052 | 0.583 |
|  | wealth | 1.051 | 0.278 | 0.292 | 0.682 | 0.003 |
|  | region | -0.516 | -0.153 | 0.079 | 0.184 | 0.014 |

**Appendix table A6. Egypt 2008. Decomposition of CI for selected pro-rich interventions**

|  |  | Egypt 2008 | | | | |
| --- | --- | --- | --- | --- | --- | --- |
|  |  | elasticities | CI | contribution to CI | contribution to CI (%) | p values |
| skilled delivery | urban | 0.009 | 0.412 | 0.004 | 0.037 | 0.058 |
|  | education | 0.071 | 0.313 | 0.022 | 0.214 | 0.000 |
|  | wealth | 0.233 | 0.278 | 0.065 | 0.623 | 0.000 |
|  | region | -0.095 | -0.168 | 0.016 | 0.155 | 0.000 |
| delivery at private secondary | urban | 0.146 | 0.412 | 0.060 | 0.119 | 0.000 |
|  | education | 0.232 | 0.313 | 0.073 | 0.144 | 0.000 |
|  | wealth | 1.017 | 0.278 | 0.283 | 0.559 | 0.000 |
|  | region | -0.136 | -0.168 | 0.023 | 0.045 | 0.000 |
| diarrhoea treatment private secondary | urban | 0.079 | 0.413 | 0.033 | 0.053 | 0.094 |
|  | education | 0.248 | 0.312 | 0.077 | 0.124 | 0.005 |
|  | wealth | 0.079 | 0.278 | 0.022 | 0.035 | 0.609 |
|  | region | -0.237 | -0.169 | 0.040 | 0.064 | 0.009 |
| cough treatment private secondary | urban | 0.222 | 0.412 | 0.092 | 0.193 | 0.016 |
|  | education | 0.316 | 0.312 | 0.099 | 0.208 | 0.115 |
|  | wealth | 0.342 | 0.278 | 0.095 | 0.200 | 0.289 |
|  | region | -0.327 | -0.169 | 0.055 | 0.116 | 0.119 |

**Appendix table A7. Egypt 2014. Decomposition of CI for selected pro-rich interventions**

|  |  | Egypt 2014 | | | | |
| --- | --- | --- | --- | --- | --- | --- |
|  |  | elasticities | CI | contribution to CI | contribution to CI (%) | p values |
| skilled delivery | urban | -0.002 | 0.565 | -0.001 | -0.033 | 0.363 |
|  | education | 0.046 | 0.191 | 0.009 | 0.241 | 0.000 |
|  | wealth | 0.072 | 0.276 | 0.020 | 0.544 | 0.000 |
|  | region | -0.049 | -0.143 | 0.007 | 0.194 | 0.000 |
| delivery at private secondary | urban | -0.035 | 0.565 | -0.020 | -0.065 | 0.058 |
|  | education | 0.349 | 0.190 | 0.066 | 0.218 | 0.000 |
|  | wealth | 0.727 | 0.276 | 0.201 | 0.660 | 0.000 |
|  | region | -0.538 | -0.143 | 0.077 | 0.253 | 0.000 |
|  |  |  |  |  |  |  |
| diarrhoea treatment public secondary | urban | 0.237 | 0.564 | 0.133 | 0.531 | 0.030 |
|  | education | -0.308 | 0.191 | -0.059 | -0.234 | 0.044 |
|  | wealth | 0.822 | 0.276 | 0.227 | 0.904 | 0.007 |
|  | region | 0.333 | -0.142 | -0.047 | -0.189 | 0.127 |
|  |  |  |  |  |  |  |
| diarrhoea treatment private secondary | urban | -0.001 | 0.566 | -0.001 | -0.001 | 0.989 |
|  | education | 0.564 | 0.191 | 0.108 | 0.210 | 0.000 |
|  | wealth | 0.420 | 0.276 | 0.116 | 0.226 | 0.194 |
|  | region | -0.634 | -0.144 | 0.091 | 0.178 | 0.006 |
| cough treatment public secondary | urban | 0.329 | 0.565 | 0.186 | 1.524 | 0.000 |
|  | education | -0.270 | 0.191 | -0.052 | -0.424 | 0.011 |
|  | wealth | 0.136 | 0.276 | 0.038 | 0.307 | 0.558 |
|  | region | 0.182 | -0.143 | -0.026 | -0.214 | 0.197 |
| cough treatment private secondary | urban | 0.183 | 0.565 | 0.103 | 0.230 | 0.041 |
|  | education | 0.468 | 0.190 | 0.089 | 0.199 | 0.005 |
|  | wealth | 0.354 | 0.276 | 0.098 | 0.218 | 0.169 |
|  | region | -0.539 | -0.143 | 0.077 | 0.172 | 0.010 |

**Appendix table A8. Jordan 2002. Decomposition of CI for selected pro-rich interventions**

|  |  | Jordan 2002 | | | | |
| --- | --- | --- | --- | --- | --- | --- |
|  |  | elasticities | CI | contribution to CI | contribution to CI (%) | p values |
| skilled delivery | urban | 0.006 | 0.041 | 0.000 | 0.035 | 0.052 |
|  | education | 0.019 | 0.046 | 0.001 | 0.125 | 0.000 |
|  | wealth | 0.017 | 0.230 | 0.004 | 0.544 | 0.003 |
|  | region | -0.001 | -0.029 | 0.000 | 0.004 | 0.764 |
| delivery at public primary | urban | -0.924 | 0.041 | -0.038 | -0.390 | 0.000 |
|  | education | 1.040 | 0.046 | 0.048 | 0.496 | 0.000 |
|  | wealth | -0.056 | 0.230 | -0.013 | -0.133 | 0.719 |
|  | region | -1.745 | -0.029 | 0.051 | 0.524 | 0.000 |
| delivery at private secondary | urban | 0.366 | 0.041 | 0.015 | 0.051 | 0.000 |
|  | education | 0.349 | 0.046 | 0.016 | 0.055 | 0.000 |
|  | wealth | 1.061 | 0.230 | 0.244 | 0.832 | 0.000 |
|  | region | -0.656 | -0.030 | 0.020 | 0.068 | 0.000 |
| diarrhoea treatment private secondary | urban | 0.234 | 0.042 | 0.010 | 0.054 | 0.356 |
|  | education | -0.118 | 0.047 | -0.006 | -0.030 | 0.744 |
|  | wealth | 0.541 | 0.230 | 0.124 | 0.683 | 0.281 |
|  | region | -0.586 | -0.030 | 0.018 | 0.097 | 0.094 |
| cough treatment private secondary | urban | 0.268 | 0.041 | 0.011 | 0.045 | 0.304 |
|  | education | -0.042 | 0.046 | -0.002 | -0.008 | 0.905 |
|  | wealth | 0.762 | 0.230 | 0.175 | 0.721 | 0.053 |
|  | region | -0.139 | -0.030 | 0.004 | 0.017 | 0.661 |

**Appendix table A9. Jordan 2007. Decomposition of CI for selected pro-rich interventions**

|  |  | Jordan 2007 | | | | |
| --- | --- | --- | --- | --- | --- | --- |
|  |  |  |  |  |  |  |
|  |  | elasticities | CI | contribution to CI | contribution to CI (%) | p values |
| skilled delivery | urban | 0.000 | 0.050 | 0.000 | 0.000 | 0.995 |
|  | education | 0.011 | 0.074 | 0.001 | 0.274 | 0.000 |
|  | wealth | 0.010 | 0.263 | 0.003 | 0.882 | 0.000 |
|  | region | 0.001 | -0.054 | 0.000 | -0.011 | 0.736 |
| delivery at public primary | urban | -0.591 | 0.051 | -0.030 | -0.364 | 0.070 |
|  | education | -0.773 | 0.074 | -0.057 | -0.701 | 0.094 |
|  | wealth | 0.250 | 0.263 | 0.066 | 0.803 | 0.561 |
|  | region | -1.396 | -0.054 | 0.076 | 0.922 | 0.000 |
| delivery at private secondary | urban | 0.246 | 0.051 | 0.012 | 0.038 | 0.000 |
|  | education | 0.519 | 0.074 | 0.038 | 0.117 | 0.000 |
|  | wealth | 1.169 | 0.263 | 0.308 | 0.933 | 0.000 |
|  | region | -0.408 | -0.054 | 0.022 | 0.067 | 0.000 |
| diarrhoea treatment private secondary | urban | 0.138 | 0.051 | 0.007 | 0.027 | 0.283 |
|  | education | -0.078 | 0.074 | -0.006 | -0.023 | 0.774 |
|  | wealth | 0.886 | 0.263 | 0.233 | 0.904 | 0.000 |
|  | region | -0.409 | -0.054 | 0.022 | 0.085 | 0.015 |
| cough treatment private secondary | urban | -0.002 | 0.050 | 0.000 | 0.000 | 0.986 |
|  | education | 0.334 | 0.074 | 0.025 | 0.079 | 0.248 |
|  | wealth | 1.010 | 0.263 | 0.266 | 0.853 | 0.000 |
|  | region | -0.440 | -0.053 | 0.023 | 0.075 | 0.002 |

**Appendix table A10. Jordan 2012. Decomposition of CI for selected pro-rich interventions**

|  |  | Jordan 2012 | | | | |
| --- | --- | --- | --- | --- | --- | --- |
|  |  | elasticities | CI | contribution to CI | contribution to CI (%) | p values |
| skilled delivery | urban | -0.001 | 0.042 | 0.000 | -0.050 | 0.118 |
|  | education | 0.003 | 0.074 | 0.000 | 0.237 | 0.046 |
|  | wealth | 0.004 | 0.273 | 0.001 | 1.031 | 0.243 |
|  | region | 0.000 | -0.032 | 0.000 | -0.006 | 0.897 |
| delivery at private secondary | urban | 0.308 | 0.042 | 0.013 | 0.043 | 0.000 |
|  | education | -0.036 | 0.073 | -0.003 | -0.009 | 0.686 |
|  | wealth | 1.155 | 0.273 | 0.316 | 1.035 | 0.000 |
|  | region | -0.334 | -0.032 | 0.011 | 0.035 | 0.000 |
| diarrhoea treatment private secondary | urban | -0.128 | 0.042 | -0.005 | -0.027 | 0.397 |
|  | education | 0.293 | 0.073 | 0.021 | 0.106 | 0.387 |
|  | wealth | 0.648 | 0.273 | 0.177 | 0.877 | 0.008 |
|  | region | -0.451 | -0.032 | 0.014 | 0.071 | 0.017 |
|  |  |  |  |  |  |  |
| cough treatment private secondary | urban | 0.101 | 0.042 | 0.004 | 0.028 | 0.420 |
|  | education | 0.820 | 0.073 | 0.060 | 0.391 | 0.006 |
|  | wealth | 0.337 | 0.273 | 0.092 | 0.598 | 0.084 |
|  | region | -0.514 | -0.033 | 0.017 | 0.109 | 0.001 |

**Appendix table A11. Jordan 2017-18. Decomposition of CI for selected pro-rich interventions**

|  |  | Jordan 2017-18 | | | | |
| --- | --- | --- | --- | --- | --- | --- |
|  |  |  |  |  |  |  |
|  |  | elasticities | CI | contribution to CI | contribution to CI (%) | p values |
| skilled delivery | urban | -0.001 | 0.035 | 0.000 | -0.050 | 0.016 |
|  | education | 0.004 | 0.066 | 0.000 | 0.314 | 0.018 |
|  | wealth | 0.001 | 0.274 | 0.000 | 0.410 | 0.209 |
|  | region | 0.000 | -0.053 | 0.000 | 0.018 | 0.752 |
| delivery at private secondary | urban | 0.338 | 0.035 | 0.012 | 0.052 | 0.000 |
|  | education | 0.167 | 0.066 | 0.011 | 0.049 | 0.040 |
|  | wealth | 0.724 | 0.274 | 0.198 | 0.874 | 0.000 |
|  | region | -0.599 | -0.053 | 0.032 | 0.141 | 0.000 |
| diarrhoea treatment private secondary | urban | 0.381 | 0.035 | 0.013 | 0.055 | 0.272 |
|  | education | 0.851 | 0.066 | 0.056 | 0.234 | 0.235 |
|  | wealth | 0.530 | 0.274 | 0.145 | 0.605 | 0.201 |
|  | region | -0.386 | -0.053 | 0.020 | 0.085 | 0.322 |
|  |  |  |  |  |  |  |
| cough treatment private secondary | urban | 0.590 | 0.035 | 0.021 | 0.068 | 0.001 |
|  | education | 1.135 | 0.066 | 0.075 | 0.245 | 0.035 |
|  | wealth | 0.685 | 0.274 | 0.188 | 0.613 | 0.023 |
|  | region | -0.180 | -0.053 | 0.010 | 0.031 | 0.476 |

**Appendix table A12. Lebanon 2011 (Palestinian refugees in Lebanon). Decomposition of CI for selected pro-rich interventions**

|  |  | Lebanon 2011 | | | | |
| --- | --- | --- | --- | --- | --- | --- |
|  |  |  |  |  |  |  |
|  |  | elasticities | CI | contribution to CI | contribution to CI (%) | p values |
| skilled delivery | urban | 0.006 | 0.057 | 0.000 | 0.334 | 0.236 |
|  | education | -0.010 | 0.046 | 0.000 | -0.477 | 0.327 |
|  | wealth | 0.006 | 0.261 | 0.002 | 1.506 | 0.419 |
|  | region | 0.002 | -0.023 | 0.000 | -0.037 | 0.749 |
| delivery at public secondary | urban | 0.195 | 0.060 | 0.012 | 0.111 | 0.038 |
|  | education | -0.365 | 0.044 | -0.016 | -0.153 | 0.243 |
|  | wealth | 0.479 | 0.261 | 0.125 | 1.178 | 0.003 |
|  | region | 0.593 | -0.022 | -0.013 | -0.125 | 0.000 |
| delivery at private secondary | urban | -0.167 | 0.060 | -0.010 | -0.050 | 0.060 |
|  | education | 1.144 | 0.046 | 0.052 | 0.260 | 0.000 |
|  | wealth | 0.672 | 0.261 | 0.175 | 0.873 | 0.000 |
|  | region | 0.454 | -0.021 | -0.009 | -0.047 | 0.000 |
| cough treatment public secondary | urban | -0.272 | 0.054 | -0.015 | -0.071 | 0.498 |
|  | education | -1.995 | 0.044 | -0.088 | -0.421 | 0.037 |
|  | wealth | 0.724 | 0.260 | 0.188 | 0.900 | 0.308 |
|  | region | 0.840 | 0.003 | 0.002 | 0.012 | 0.022 |
|  |  |  |  |  |  |  |
| cough treatment private secondary | urban | -0.017 | 0.057 | -0.001 | -0.005 | 0.914 |
|  | education | 0.511 | 0.045 | 0.023 | 0.116 | 0.261 |
|  | wealth | 0.232 | 0.260 | 0.060 | 0.305 | 0.443 |
|  | region | -0.565 | 0.003 | -0.002 | -0.009 | 0.025 |

**Appendix table A13. Tunisia 2012. Decomposition of CI for selected pro-rich interventions**

|  |  | Tunisia 2012 | | | | |
| --- | --- | --- | --- | --- | --- | --- |
|  |  |  |  |  |  |  |
|  |  | elasticities | CI | contribution to CI | contribution to CI (%) | p values |
| skilled delivery | urban | 0.001 | 0.232 | 0.000 | 0.035 | 0.154 |
|  | education | 0.005 | 0.092 | 0.001 | 0.072 | 0.002 |
|  | wealth | 0.004 | 0.263 | 0.001 | 0.163 | 0.162 |
|  | region | -0.003 | -0.103 | 0.000 | 0.037 | 0.007 |
| delivery at private secondary | urban | 0.033 | 0.231 | 0.008 | 0.013 | 0.753 |
|  | education | 0.934 | 0.093 | 0.087 | 0.150 | 0.000 |
|  | wealth | 1.151 | 0.263 | 0.303 | 0.526 | 0.000 |
|  | region | -0.221 | -0.104 | 0.023 | 0.040 | 0.000 |
| cough treatment private secondary | urban | -0.276 | 0.229 | -0.063 | -0.197 | 0.071 |
|  | education | 1.288 | 0.121 | 0.156 | 0.486 | 0.001 |
|  | wealth | 0.864 | 0.263 | 0.227 | 0.707 | 0.004 |
|  | region | -0.009 | -0.095 | 0.001 | 0.003 | 0.950 |

**Appendix table A14. Tunisia 2018. Decomposition of CI for selected pro-rich interventions**

|  |  | Tunisia 2018 | | | | |
| --- | --- | --- | --- | --- | --- | --- |
|  |  |  |  |  |  |  |
|  |  | elasticities | CI | contribution to CI | contribution to CI (%) | p values |
| skilled delivery | urban | 0.000 | 0.236 | 0.000 | -0.002 | 0.729 |
|  | education | 0.000 | 0.114 | 0.000 | 0.015 | 0.000 |
|  | wealth | 0.000 | 0.264 | 0.000 | 0.013 | 0.396 |
|  | region | 0.000 | -0.130 | 0.000 | 0.011 | 0.000 |
| delivery at public primary | urban | -0.481 | 0.235 | -0.113 | -1.109 | 0.164 |
|  | education | 0.036 | 0.114 | 0.004 | 0.040 | 0.934 |
|  | wealth | 0.870 | 0.264 | 0.230 | 2.250 | 0.128 |
|  | region | 0.181 | -0.129 | -0.023 | -0.229 | 0.642 |
| delivery at private secondary | urban | -0.092 | 0.237 | -0.022 | -0.048 | 0.371 |
|  | education | 0.691 | 0.115 | 0.079 | 0.176 | 0.000 |
|  | wealth | 1.475 | 0.264 | 0.389 | 0.864 | 0.000 |
|  | region | -0.127 | -0.130 | 0.016 | 0.037 | 0.177 |
| diarrhoea treatment private primary | urban | 0.086 | 0.263 | 0.023 | 0.139 | 0.700 |
|  | education | 0.262 | 0.138 | 0.036 | 0.223 | 0.390 |
|  | wealth | 0.291 | 0.263 | 0.077 | 0.470 | 0.487 |
|  | region | -0.488 | -0.103 | 0.050 | 0.309 | 0.071 |
| cough treatment private secondary | urban | 0.231 | 0.261 | 0.060 | 0.158 | 0.414 |
|  | education | -0.649 | 0.139 | 0.090 | -0.236 | 0.136 |
|  | wealth | 1.456 | 0.263 | 0.383 | 1.003 | 0.012 |
|  | region | 0.310 | -0.102 | -0.032 | -0.083 | 0.414 |
| cough treatment private primary | urban | 0.018 | 0.259 | 0.005 | 0.021 | 0.879 |
|  | education | 0.387 | 0.139 | 0.054 | 0.240 | 0.017 |
|  | wealth | 0.630 | 0.263 | 0.166 | 0.740 | 0.003 |
|  | region | -0.142 | -0.102 | 0.014 | 0.064 | 0.339 |

**Appendix Table A15. Iraq 2011: Decomposition of CI for selected pro-rich interventions**

|  |  | Iraq 2011 | | | | |
| --- | --- | --- | --- | --- | --- | --- |
|  |  | elasticities | CI | contribution to CI | contribution to CI (%) | p values |
| skilled delivery | urban | 0.032 | 0.162 | 0.005 | 0.171 | 0.000 |
|  | education | 0.097 | 0.083 | 0.008 | 0.268 | 0.000 |
|  | wealth | 0.063 | 0.259 | 0.016 | 0.541 | 0.000 |
|  | region | -0.047 | 0.004 | 0.000 | -0.006 | 0.000 |
| delivery at private secondary | urban | 0.064 | 0.162 | 0.010 | 0.026 | 0.306 |
|  | education | 1.048 | 0.083 | 0.087 | 0.220 | 0.000 |
|  | wealth | 1.047 | 0.259 | 0.271 | 0.690 | 0.000 |
|  | region | -0.402 | 0.004 | -0.002 | -0.004 | 0.059 |
| cough treatment private secondary | urban | -0.110 | 0.182 | -0.020 | -0.298 | 0.570 |
|  | education | 0.983 | 0.080 | 0.079 | 1.178 | 0.019 |
|  | wealth | 0.062 | 0.282 | 0.017 | 0.259 | 0.856 |
|  | region | -0.554 | 0.003 | -0.002 | -0.029 | 0.223 |
| cough treatment private primary | urban | 0.027 | 0.182 | 0.005 | 0.033 | 0.619 |
|  | education | 0.067 | 0.080 | 0.005 | 0.036 | 0.589 |
|  | wealth | 0.535 | 0.282 | 0.151 | 1.012 | 0.000 |
|  | region | 0.617 | 0.003 | 0.002 | 0.013 | 0.000 |

**Appendix Table A16. Iraq 2018: Decomposition of CI for selected pro-rich interventions**

|  |  | Iraq 2018 | | | | |
| --- | --- | --- | --- | --- | --- | --- |
|  |  |  |  |  |  |  |
|  |  | elasticities | CI | contribution to CI | contribution to CI (%) | p values |
| skilled delivery | urban | 0.016 | 0.137 | 0.002 | 0.205 | 0.001 |
|  | education | 0.017 | 0.124 | 0.002 | 0.191 | 0.020 |
|  | wealth | 0.018 | 0.258 | 0.005 | 0.424 | 0.116 |
|  | region | -0.041 | -0.057 | 0.002 | 0.211 | 0.116 |
| delivery at private secondary | urban | -0.042 | 0.133 | -0.006 | -0.015 | 0.774 |
|  | education | 0.387 | 0.125 | 0.049 | 0.132 | 0.000 |
|  | wealth | 1.053 | 0.258 | 0.272 | 0.741 | 0.000 |
|  | region | -0.487 | -0.058 | 0.028 | 0.077 | 0.108 |
| diarrhoea treatment public secondary | urban | 0.068 | 0.142 | 0.010 | 0.055 | 0.568 |
|  | education | -0.366 | 0.149 | -0.054 | -0.309 | 0.008 |
|  | wealth | 0.782 | 0.278 | 0.217 | 1.233 | 0.010 |
|  | region | -0.175 | -0.052 | 0.009 | 0.052 | 0.760 |
|  |  |  |  |  |  |  |
| diarrhoea treatment private primary | urban | -0.064 | 0.144 | -0.009 | -0.179 | 0.452 |
|  | education | 0.158 | 0.149 | 0.024 | 0.462 | 0.066 |
|  | wealth | -0.045 | 0.278 | -0.012 | -0.243 | 0.799 |
|  | region | -1.235 | -0.051 | 0.064 | 1.247 | 0.001 |
| cough treatment private primary | urban | -0.161 | 0.143 | -0.023 | -0.275 | 0.065 |
|  | education | 0.157 | 0.149 | 0.023 | 0.279 | 0.054 |
|  | wealth | -0.061 | 0.278 | -0.017 | -0.200 | 0.674 |
|  | region | -1.730 | -0.052 | 0.090 | 1.075 | 0.000 |

**Appendix Table A17. Morocco 2003: Decomposition of CI for selected pro-rich interventions**

|  |  | Morocco 2003-04 | | | | |
| --- | --- | --- | --- | --- | --- | --- |
|  |  |  |  |  |  |  |
|  |  | elasticities | CI | contribution to CI | contribution to CI (%) | p values |
| skilled delivery | urban | 0.178 | 0.420 | 0.075 | 0.346 | 0.000 |
|  | education | 0.090 | 0.517 | 0.046 | 0.215 | 0.000 |
|  | wealth | 0.437 | 0.281 | 0.123 | 0.567 | 0.000 |
|  | region | -0.071 | 0.005 | 0.000 | -0.002 | 0.000 |
| delivery at public secondary | urban | 0.349 | 0.422 | 0.147 | 0.722 | 0.000 |
|  | education | 0.003 | 0.518 | 0.002 | 0.009 | 0.697 |
|  | wealth | 0.213 | 0.281 | 0.060 | 0.293 | 0.000 |
|  | region | 0.026 | 0.005 | 0.000 | 0.001 | 0.507 |
| delivery at private secondary | urban | 0.022 | 0.420 | 0.009 | 0.013 | 0.513 |
|  | education | 0.077 | 0.517 | 0.040 | 0.057 | 0.000 |
|  | wealth | 0.821 | 0.281 | 0.231 | 0.329 | 0.000 |
|  | region | 0.061 | 0.003 | 0.000 | 0.000 | 0.182 |
| diarrhoea treatment public secondary | urban | 0.716 | 0.421 | 0.301 | 0.997 | 0.029 |
|  | education | -0.133 | 0.520 | -0.069 | -0.229 | 0.132 |
|  | wealth | 0.342 | 0.281 | 0.096 | 0.318 | 0.576 |
|  | region | -0.279 | 0.006 | -0.002 | -0.005 | 0.605 |
| cough treatment public secondary | urban | 0.375 | 0.421 | 0.158 | 0.828 | 0.030 |
|  | education | -0.032 | 0.518 | -0.017 | -0.088 | 0.552 |
|  | wealth | 0.248 | 0.281 | 0.070 | 0.364 | 0.490 |
|  | region | 0.293 | 0.004 | 0.001 | 0.006 | 0.337 |

**Appendix Table A18. Morocco 2011: Decomposition of CI for selected pro-rich interventions**

|  |  | Morocco 2011 | | | | |
| --- | --- | --- | --- | --- | --- | --- |
|  |  | elasticities | CI | contribution to CI | contribution to CI (%) | p values |
| skilled delivery | urban |  |  |  |  |  |
|  | education | 0.181 | 0.407 | 0.074 | 0.102 | 0.003 |
|  | wealth | 1.340 | 0.263 | 0.353 | 0.490 | 0.000 |
|  | region | -0.144 | 0.011 | -0.002 | -0.002 | 0.400 |
| delivery at public secondary | urban |  |  |  |  |  |
|  | education | -0.046 | 0.406 | -0.019 | -0.156 | 0.001 |
|  | wealth | 0.527 | 0.263 | 0.139 | 1.166 | 0.000 |
|  | region | -0.015 | 0.011 | 0.000 | -0.001 | 0.669 |
| delivery at private primary | urban |  |  |  |  |  |
|  | education | 0.241 | 0.407 | 0.098 | 0.159 | 0.000 |
|  | wealth | 1.071 | 0.263 | 0.282 | 0.456 | 0.000 |
|  | region | 0.082 | 0.011 | 0.001 | 0.002 | 0.196 |
| diarrhoea treatment public secondary | urban |  |  |  |  |  |
|  | education | -0.047 | 0.407 | 0.019 | -0.232 | 0.768 |
|  | wealth | 0.378 | 0.263 | 0.100 | 1.214 | 0.525 |
|  | region | 1.091 | 0.010 | 0.011 | 0.134 | 0.034 |
| diarrhoea treatment private primary | urban |  |  |  |  |  |
|  | education | 0.193 | 0.406 | 0.078 | 0.233 | 0.109 |
|  | wealth | 1.021 | 0.263 | 0.269 | 0.798 | 0.045 |
|  | region | 0.202 | 0.009 | 0.002 | 0.006 | 0.575 |
| cough treatment public secondary | urban |  |  |  |  |  |
|  | education | 0.020 | 0.407 | 0.008 | 0.049 | 0.814 |
|  | wealth | 0.672 | 0.263 | 0.177 | 1.085 | 0.016 |
|  | region | -0.293 | 0.010 | -0.003 | -0.019 | 0.282 |
| cough treatment private primary | urban |  |  |  |  |  |
|  | education | 0.278 | 0.406 | 0.113 | 0.371 | 0.000 |
|  | wealth | 0.449 | 0.263 | 0.118 | 0.389 | 0.011 |
|  | region | 0.016 | 0.009 | 0.000 | 0.001 | 0.914 |

**Appendix Table A19. Morocco 2018: Decomposition of CI for selected pro-rich interventions**

|  |  | Morocco 2018 | | | | |
| --- | --- | --- | --- | --- | --- | --- |
|  |  | elasticities | CI | contribution to CI | contribution to CI (%) | p values |
| skilled delivery | urban | 0.072 | 0.314 | 0.022 | 0.321 | 0.000 |
|  | education | 0.072 | 0.220 | 0.016 | 0.225 | 0.000 |
|  | wealth | 0.091 | 0.268 | 0.024 | 0.348 | 0.000 |
|  | region | 0.009 | 0.006 | 0.000 | 0.001 | 0.353 |
|  |  |  |  |  |  |  |
| delivery at public secondary | urban | 0.157 | 0.314 | 0.049 | 1.176 | 0.000 |
|  | education | -0.116 | 0.219 | -0.026 | -0.608 | 0.000 |
|  | wealth | 0.066 | 0.268 | 0.018 | 0.424 | 0.100 |
|  | region | -0.026 | 0.006 | 0.000 | -0.004 | 0.321 |
| delivery at private primary | urban | 0.263 | 0.314 | 0.083 | 0.216 | 0.000 |
|  | education | 0.526 | 0.219 | 0.115 | 0.302 | 0.000 |
|  | wealth | 0.542 | 0.268 | 0.145 | 0.381 | 0.000 |
|  | region | -0.191 | 0.006 | -0.001 | -0.003 | 0.000 |
| diarrhoea treatment private primary | urban | 0.288 | 0.314 | 0.090 | 0.584 | 0.123 |
|  | education | 0.235 | 0.219 | 0.052 | 0.333 | 0.126 |
|  | wealth | -0.027 | 0.268 | -0.007 | -0.046 | 0.940 |
|  | region | 0.070 | 0.006 | 0.000 | 0.003 | 0.763 |
|  |  |  |  |  |  |  |
| cough treatment public secondary | urban | 0.027 | 0.314 | 0.008 | 0.067 | 0.916 |
|  | education | -0.083 | 0.219 | -0.018 | -0.143 | 0.467 |
|  | wealth | 0.556 | 0.268 | 0.149 | 1.173 | 0.148 |
|  | region | -0.275 | 0.006 | -0.002 | -0.013 | 0.197 |
| cough treatment private primary | urban | 0.110 | 0.314 | 0.035 | 0.189 | 0.329 |
|  | education | 0.317 | 0.219 | 0.070 | 0.380 | 0.000 |
|  | wealth | 0.263 | 0.268 | 0.070 | 0.385 | 0.202 |
|  | region | -0.072 | 0.006 | 0.000 | -0.002 | 0.577 |

**Appendix Table A20. State of Palestine 2010: Decomposition of CI for selected pro-rich interventions**

|  |  | Palestine 2010 | | | | |
| --- | --- | --- | --- | --- | --- | --- |
|  |  |  |  |  |  |  |
|  |  | elasticities | CI | contribution to CI | contribution to CI (%) | p values |
| delivery at public primary | urban | -0.374 | 0.047 | -0.018 | -0.255 | 0.155 |
|  | education | -0.144 | 0.045 | -0.007 | -0.094 | 0.794 |
|  | wealth | 0.362 | 0.260 | 0.094 | 1.366 | 0.352 |
|  | region | -0.097 | -0.045 | 0.004 | 0.064 | 0.829 |
| delivery at private secondary | urban | 0.046 | 0.046 | 0.002 | 0.011 | 0.282 |
|  | education | 0.539 | 0.044 | 0.024 | 0.119 | 0.000 |
|  | wealth | 0.572 | 0.260 | 0.149 | 0.740 | 0.000 |
|  | region | -0.895 | -0.045 | 0.041 | 0.202 | 0.000 |
| cough treatment private secondary | urban | -0.310 | 0.040 | -0.013 | -0.119 | 0.230 |
|  | education | 0.479 | 0.045 | 0.022 | 0.205 | 0.473 |
|  | wealth | 0.242 | 0.275 | 0.067 | 0.634 | 0.535 |
|  | region | -0.272 | -0.046 | 0.012 | 0.119 | 0.367 |
| cough treatment private primary | urban | 0.104 | 0.041 | 0.004 | 0.022 | 0.267 |
|  | education | 0.101 | 0.045 | 0.005 | 0.023 | 0.709 |
|  | wealth | 0.523 | 0.275 | 0.144 | 0.742 | 0.000 |
|  | region | -1.039 | -0.039 | 0.040 | 0.207 | 0.000 |

**Appendix Table A21. State of Palestine 2014: Decomposition of CI for selected pro-rich interventions**

|  |  | Palestine 2014 | | | | |
| --- | --- | --- | --- | --- | --- | --- |
|  |  |  |  |  |  |  |
|  |  | elasticities | CI | contribution to CI | contribution to CI (%) | p values |
| skilled delivery | urban | 0.002 | -0.014 | 0.000 | -0.003 | 0.328 |
|  | education | 0.001 | 0.018 | 0.000 | 0.004 | 0.569 |
|  | wealth | 0.000 | 0.264 | 0.000 | 0.008 | 0.942 |
|  | region | -0.004 | -0.155 | 0.001 | 0.087 | 0.356 |
| delivery at private secondary | urban | 0.071 | -0.013 | -0.001 | -0.003 | 0.211 |
|  | education | 0.631 | 0.018 | 0.011 | 0.042 | 0.000 |
|  | wealth | 0.802 | 0.264 | 0.212 | 0.793 | 0.000 |
|  | region | -0.267 | -0.155 | 0.041 | 0.155 | 0.099 |
| diarrhoea treatment private secondary | urban | -0.513 | -0.023 | 0.012 | 0.030 | 0.026 |
|  | education | 1.339 | 0.026 | 0.034 | 0.089 | 0.001 |
|  | wealth | 1.247 | 0.283 | 0.353 | 0.914 | 0.001 |
|  | region | 0.408 | -0.156 | -0.064 | -0.165 | 0.503 |
| diarrhoea treatment private primary | urban | -0.016 | -0.025 | 0.000 | 0.001 | 0.896 |
|  | education | 0.478 | 0.025 | 0.012 | 0.034 | 0.093 |
|  | wealth | 0.817 | 0.283 | 0.231 | 0.672 | 0.001 |
|  | region | -0.743 | -0.156 | 0.116 | 0.337 | 0.060 |
|  | region |  |  |  |  |  |
| cough treatment private secondary | urban | -0.078 | -0.025 | 0.002 | 0.007 | 0.221 |
|  | education | 0.623 | 0.025 | 0.015 | 0.054 | 0.000 |
|  | wealth | 0.327 | 0.283 | 0.093 | 0.325 | 0.012 |
|  | region | -1.235 | -0.156 | 0.193 | 0.677 | 0.000 |
|  |  |  |  |  |  |  |
| cough treatment private primary | urban | 0.115 | -0.026 | -0.003 | -0.010 | 0.449 |
|  | education | 0.303 | 0.025 | 0.008 | 0.027 | 0.370 |
|  | wealth | 0.803 | 0.283 | 0.227 | 0.789 | 0.010 |
|  | region | -0.266 | -0.155 | 0.041 | 0.143 | 0.594 |

**Appendix table A22. Sudan 2010: Decomposition of CI for selected pro-rich interventions**

|  |  | Sudan 2010 | | | | |
| --- | --- | --- | --- | --- | --- | --- |
|  |  |  |  |  |  |  |
|  |  | elasticities | CI | contribution to CI | contribution to CI (%) | p values |
| skilled delivery | urban | 0.030 | 0.406 | 0.012 | 0.099 | 0.000 |
|  | education | 0.137 | 0.128 | 0.018 | 0.142 | 0.000 |
|  | wealth | 0.362 | 0.255 | 0.092 | 0.745 | 0.000 |
|  | region | -0.141 | -0.094 | 0.013 | 0.107 | 0.000 |
| diarrhoea treatment private secondary | urban | 0.338 | 0.464 | 0.157 | 0.412 | 0.010 |
|  | education | 0.038 | 0.102 | 0.004 | 0.010 | 0.854 |
|  | wealth | 0.823 | 0.280 | 0.230 | 0.604 | 0.037 |
|  | region | 0.668 | -0.094 | -0.063 | -0.165 | 0.114 |
|  |  |  |  |  |  |  |
| diarrhoea treatment private primary | urban | -0.034 | 0.464 | -0.016 | -0.083 | 0.660 |
|  | education | 0.141 | 0.100 | 0.014 | 0.075 | 0.330 |
|  | wealth | 0.965 | 0.280 | 0.270 | 1.444 | 0.000 |
|  | region | 0.782 | -0.095 | -0.074 | -0.396 | 0.015 |
| cough treatment public secondary | urban | 0.080 | 0.466 | 0.037 | 0.390 | 0.011 |
|  | education | -0.177 | 0.101 | -0.018 | -0.187 | 0.024 |
|  | wealth | 0.111 | 0.280 | 0.031 | 0.323 | 0.341 |
|  | region | -0.429 | -0.096 | 0.041 | 0.431 | 0.001 |
| cough treatment private secondary | urban | 0.369 | 0.467 | 0.173 | 0.458 | 0.000 |
|  | education | -0.256 | 0.102 | -0.026 | -0.069 | 0.239 |
|  | wealth | 0.698 | 0.280 | 0.195 | 0.518 | 0.026 |
|  | region | 0.498 | -0.097 | -0.048 | -0.127 | 0.100 |
|  |  |  |  |  |  |  |
| cough treatment private primary | urban | 0.093 | 0.463 | 0.043 | 0.180 | 0.065 |
|  | education | 0.179 | 0.100 | 0.018 | 0.076 | 0.080 |
|  | wealth | 0.864 | 0.280 | 0.242 | 1.015 | 0.000 |
|  | region | 0.884 | -0.096 | -0.085 | -0.356 | 0.000 |

**Appendix table A23. Sudan 2014: Decomposition of CI for selected pro-rich interventions**

|  |  | Sudan 2014 | | | | |
| --- | --- | --- | --- | --- | --- | --- |
|  |  |  |  |  |  |  |
|  |  | elasticities | CI | contribution to CI | contribution to CI (%) | p values |
| skilled delivery | urban | 0.028 | 0.378 | 0.011 | 0.083 | 0.000 |
|  | education | 0.283 | 0.153 | 0.043 | 0.335 | 0.000 |
|  | wealth | 0.271 | 0.256 | 0.069 | 0.537 | 0.000 |
|  | region | -0.200 | -0.107 | 0.021 | 0.166 | 0.000 |
| delivery at public secondary | urban | 0.041 | 0.377 | 0.015 | 0.042 | 0.087 |
|  | education | 0.851 | 0.152 | 0.129 | 0.347 | 0.000 |
|  | wealth | 0.692 | 0.256 | 0.177 | 0.476 | 0.000 |
|  | region | -0.710 | -0.106 | 0.075 | 0.203 | 0.000 |
| delivery at public primary | urban | -0.239 | 0.377 | -0.090 | -0.739 | 0.041 |
|  | education | 0.396 | 0.152 | 0.060 | 0.495 | 0.383 |
|  | wealth | 0.810 | 0.256 | 0.207 | 1.699 | 0.260 |
|  | region | 0.553 | -0.106 | -0.059 | -0.481 | 0.185 |
| delivery at private secondary | urban | 0.155 | 0.374 | 0.058 | 0.081 | 0.000 |
|  | education | 0.169 | 0.153 | 0.026 | 0.036 | 0.004 |
|  | wealth | 0.305 | 0.256 | 0.078 | 0.109 | 0.007 |
|  | region | -0.246 | -0.107 | 0.026 | 0.037 | 0.000 |
| delivery at private primary | urban | 0.014 | 0.376 | 0.005 | 0.007 | 0.036 |
|  | education | 0.005 | 0.153 | 0.001 | 0.001 | 0.777 |
|  | wealth | 0.080 | 0.256 | 0.020 | 0.025 | 0.030 |
|  | region | -0.009 | -0.106 | 0.001 | 0.001 | 0.585 |
| diarrhoea treatment private secondary | urban | 0.133 | 0.420 | 0.056 | 0.388 | 0.116 |
|  | education | 0.262 | 0.154 | 0.040 | 0.280 | 0.272 |
|  | wealth | 0.498 | 0.275 | 0.137 | 0.952 | 0.160 |
|  | region | 0.846 | -0.103 | -0.087 | -0.607 | 0.011 |
| diarrhoea treatment private primary | urban | 0.051 | 0.424 | 0.022 | 0.222 | 0.577 |
|  | education | 0.225 | 0.154 | 0.035 | 0.353 | 0.441 |
|  | wealth | -0.116 | 0.275 | -0.032 | -0.326 | 0.767 |
|  | region | -0.614 | -0.103 | 0.063 | 0.644 | 0.146 |
| cough treatment public secondary | urban | -0.030 | 0.420 | -0.012 | -0.264 | 0.294 |
|  | education | 0.201 | 0.154 | 0.031 | 0.657 | 0.046 |
|  | wealth | 0.048 | 0.275 | 0.013 | 0.282 | 0.710 |
|  | region | -0.156 | -0.103 | 0.016 | 0.342 | 0.203 |
| cough treatment private secondary | urban | 0.351 | 0.422 | 0.148 | 0.626 | 0.000 |
|  | education | -0.066 | 0.153 | -0.010 | -0.043 | 0.761 |
|  | wealth | 0.693 | 0.275 | 0.191 | 0.809 | 0.012 |
|  | region | 1.108 | -0.103 | -0.114 | -0.484 | 0.000 |
| cough treatment private primary | urban | 0.216 | 0.423 | 0.091 | 0.545 | 0.001 |
|  | education | 0.672 | 0.154 | 0.103 | 0.619 | 0.003 |
|  | wealth | -0.427 | 0.275 | -0.118 | -0.705 | 0.178 |
|  | region | -0.560 | -0.103 | 0.058 | 0.344 | 0.106 |

**Appendix table A24. Syria 2006: Decomposition of CI for selected pro-rich interventions**

|  |  | Syria2006 | | | | |
| --- | --- | --- | --- | --- | --- | --- |
|  |  |  |  |  |  |  |
|  |  | elasticities | CI | contribution to CI | contribution to CI (%) | p values |
| skilled delivery | urban | 0.014 | 0.282 | 0.004 | 0.099 | 0.000 |
|  | education | 0.093 | 0.094 | 0.009 | 0.223 | 0.000 |
|  | wealth | 0.068 | 0.262 | 0.018 | 0.458 | 0.000 |
|  | region |  |  |  |  |  |
| delivery at private secondary | urban | 0.091 | 0.281 | 0.026 | 0.091 | 0.003 |
|  | education | 0.771 | 0.094 | 0.072 | 0.256 | 0.000 |
|  | wealth | 0.813 | 0.262 | 0.213 | 0.755 | 0.000 |
|  | region |  |  |  |  |  |

**Appendix table A25. Yemen 2006: Decomposition of CI for selected pro-rich interventions**

|  |  | Yemen 2006 | | | | |
| --- | --- | --- | --- | --- | --- | --- |
|  |  |  |  |  |  |  |
|  |  | elasticities | CI | contribution to CI | contribution to CI (%) | p values |
| skilled delivery | urban | 0.118 | 0.465 | 0.055 | 0.240 | 0.000 |
|  | education | 0.145 | 0.106 | 0.015 | 0.067 | 0.090 |
|  | wealth | 0.947 | 0.254 | 0.241 | 1.052 | 0.000 |
|  | region | 0.044 | -0.015 | -0.001 | -0.003 | 0.721 |
| delivery at public secondary | urban | 0.069 | 0.465 | 0.032 | 0.084 | 0.124 |
|  | education | -0.252 | 0.107 | -0.027 | -0.071 | 0.038 |
|  | wealth | 1.416 | 0.254 | 0.360 | 0.943 | 0.000 |
|  | region | 0.323 | -0.014 | -0.005 | -0.012 | 0.066 |
| delivery at private secondary | urban | 0.191 | 0.462 | 0.088 | 0.326 | 0.039 |
|  | education | 0.196 | 0.104 | 0.020 | 0.076 | 0.331 |
|  | wealth | 0.519 | 0.254 | 0.132 | 0.489 | 0.103 |
|  | region | 0.112 | -0.014 | -0.002 | -0.006 | 0.769 |
| delivery at private primary | urban | 0.068 | 0.464 | 0.032 | 0.130 | 0.610 |
|  | education | 0.152 | 0.105 | 0.016 | 0.066 | 0.677 |
|  | wealth | 0.664 | 0.254 | 0.169 | 0.692 | 0.092 |
|  | region | -0.347 | -0.014 | 0.005 | 0.020 | 0.593 |

**Appendix table A26. Yemen 2013: Decomposition of CI for selected pro-rich interventions**

|  |  | Yemen2013 | | | | |
| --- | --- | --- | --- | --- | --- | --- |
|  |  |  |  |  |  |  |
|  |  | elasticities | CI | contribution to CI | contribution to CI (%) | p values |
| skilled delivery | urban | 0.079 | 0.579 | 0.046 | 0.170 | 0.000 |
|  | education | |  |  |  |  |
|  | wealth | 0.929 | 0.270 | 0.251 | 0.932 | 0.000 |
|  | region | -0.128 | -0.030 | 0.004 | 0.014 | 0.000 |
| delivery at public secondary | urban | 0.007 | 0.580 | 0.004 | 0.012 | 0.668 |
|  | education | |  |  |  |  |
|  | wealth | 1.161 | 0.270 | 0.313 | 0.981 | 0.000 |
|  | region | 0.014 | -0.029 | 0.000 | -0.001 | 0.829 |
| delivery at public primary | urban | 0.093 | 0.578 | 0.054 | 13.489 | 0.112 |
|  | education | |  |  |  |  |
|  | wealth | -0.194 | 0.270 | -0.052 | -13.067 | 0.275 |
|  | region | -0.122 | -0.029 | 0.004 | 0.899 | 0.543 |
| delivery at private secondary | urban | 0.006 | 0.580 | 0.003 | 0.012 | 0.780 |
|  | education | |  |  |  |  |
|  | wealth | 1.029 | 0.270 | 0.277 | 0.919 | 0.000 |
|  | region | -0.285 | -0.029 | 0.008 | 0.027 | 0.000 |
| diarrhoea treatment public secondary | urban | 0.021 | 0.580 | 0.012 | 0.097 | 0.665 |
|  | education | |  |  |  |  |
|  | wealth | 0.398 | 0.270 | 0.107 | 0.832 | 0.009 |
|  | region | -0.196 | -0.029 | 0.006 | 0.044 | 0.252 |
|  |  |  |  |  |  |  |
| diarrhoea treatment private secondary | urban | 0.097 | 0.579 | 0.056 | 0.311 | 0.014 |
|  | education | |  |  |  |  |
|  | wealth | 0.457 | 0.270 | 0.123 | 0.681 | 0.000 |
|  | region | -0.168 | -0.029 | 0.005 | 0.027 | 0.157 |
| cough treatment public secondary | urban | -0.012 | 0.580 | -0.007 | -0.059 | 0.798 |
|  | education | |  |  |  |  |
|  | wealth | 0.460 | 0.270 | 0.124 | 1.059 | 0.001 |
|  | region | 0.035 | -0.029 | -0.001 | -0.009 | 0.833 |
